# Adsorption and desorption of methyl orange dye on environmentally aged polyethylene, polyethylene terephthalate and polystyrene microplastics in aquatic environment

**DOI:** 10.1371/journal.pone.0323516

**Published:** 2025-07-28

**Authors:** Ahsan Habib, Sayedunnesa Nishi, Md. Muhaiminul Haque, Md. Tauhiduzzaman, Kobita Khatun, Mst Shamima Akter, Esrat Sultana, Tapos Kumar Chakraborty, Samina Zaman, Gopal Chandra Ghosh

**Affiliations:** Department of Environmental Science and Technology, Jashore University of Science and Technology, Jashore, Bangladesh; VIT University, INDIA

## Abstract

This study investigated the adsorption of methyl orange (MO), an anionic dye, on environmentally aged polyethylene (PE), polyethylene terephthalate (PET), and polystyrene (PS) microplastics (MPs) to understand their interactions in aquatic environments. The MPs were characterized using Fourier transform infrared spectroscopy (FTIR) and scanning electron microscopy (SEM). The adsorption experiment data followed pseudo-second-order kinetics and fit well with the Langmuir and Freundlich isotherm models. The adsorption capacities of MO onto the MPs were 2.86 mg/g, 3.64 mg/g, and 3.81 mg/g for PE, PET, and PS, respectively, at a neutral pH of 7.0. The MPs combined with MO mainly through chemisorption, hydrogen bonding, and hydrophilic interaction. The optimum conditions for MO (23.41% for PE, 22.8% for PET, and 22.64% for PS) removal by the MPs were: pH 2, MO concentration of 27.5 mg/L, and MPs dose of 15.0 g/L, as determined using response surface methodology (RSM). Additionally, the presence of salt (NaCl) and humic acid (HA) competed with MO for adsorption sites on the MPs. The desorption of MO from the MPs was relatively higher in freshwater (16–30%) than in simulated seawater (12–19%). This study elucidates the interaction of MO with environmentally aged PE, PET, and PS MPs in aquatic environments, and demonstrates the transport capacity of MO dye from wastewater to freshwater, and eventually to the ocean.

## 1. Introduction

Microplastics (MPs) are either fragments of larger plastic particles or engineered plastic particles less than 5 mm in size [1]. Over the years, MPs have emerged as contaminants of concern due to their extensive use, widespread presence, persistence, difficulty in degradation, and eco-toxicological effects [2]. Global plastic production has risen dramatically from 1.5 million tons in the 1950s to 390 million tons in 2021 [3]. The combination of continuous disposal and low recycling rates (10%) has led to significant environmental challenges [4–9]. One of these is the gradual breakdown of plastic polymers into smaller fragments, such as MPs [10,11]. Plastics are highly persistent in aquatic environments, causing harmful physical and biological consequences to organisms, including plants and animals life [12] by ingestion, entanglement, and physical damage [13–16]. During this period plastic particles undergo a range of weathering processes driven by factors such as sunlight, heat, thermal breakdown, biological activity and oxidative reactions. These processes, collectively known as *environmental aging*, can significantly modify the physical and chemical characteristics of materials—such as surface morphology, specific surface area, hydrophobicity, structural integrity, mechanical resilience, and elemental composition—and may influence their behavior in aquatic environments as well as their interactions with pollutants [17–19]. Toxic chemicals leaching from MPs, such as additives like plasticizers, flame retardants, and antioxidants, pose further risks to marine organisms by entering the food chain and triggering bioaccumulation and biomagnification [9,20–26].

The potential of MPs to transfer and expose pollutants to aquatic ecosystems is enhanced by their small size, large surface area, porosity, amorphous structure, strong hydrophobicity, and various functional groups [27–29]. MPs readily adsorb and interact with organic and inorganic contaminants [26,30,31] such as toxic trace elements [32], heavy metals [33], fungicides [34], antibiotics [35], dyes [22,36,37], persistent organic pollutants (POPs), polychlorinated biphenyls (PCBs), polyaromatic hydrocarbons (PAHs), bacteria, and viruses [21,38–40]. These interactions create secondary pollutants, amplifying toxic effects on ecological functions [31], and these complex contaminants may release pollutants upon entering new environments [41]. Organisms consuming such contaminants are exposed to dual toxicity risks [10,41–43].

Therefore polymers are increasingly recognized for their potential as effective adsorbent, largely due to their diverse physiochemical characteristics such as modifiable surface properties [36] which results affinity for adsorption, relative high surface area, functional groups [31,44] electronic structures [45–48]. Recent studies have shown that nonconductive polymers like polyethylene (PE), Polyethylene Terephthalate (PET) and polystyrene (PS) can exhibit substantial adsorption capacities for organic and inorganic contaminants [17,28,36,49,50]. The primary attributed to mechanisms such as van der Waals forces, hydrophobic interactions, and π–π interactions. Furthermore environmentally aged MPs surfaces led to the formation of oxygen containing functional groups and micro cracks, thereby enhancing the adsorption capacity of MPs with contaminants [49].

Among the toxic pollutants adsorbed by MPs, dyes are of critical concern, particularly in developing nations [51]. Dyes are widely used in industries such as plastics, textiles, cosmetics, paper, paints, food, ink, varnishes, and tanneries [52–54]. Globally, over 700,000 tons of dyes are produced annually, with the textile industry as the largest consumer [55]. During dyeing processes, 10–15% of dye contaminants are discharged into aquatic environments [49,56]. Alarmingly, over 50% of dyes used during industrial manufacturing do not adhere to fabrics and are released as colored effluent [57,58]. Even trace amounts of dyes significantly restrict light penetration in aquatic environments, disrupting photosynthesis and biological metabolism in organisms [59,60].

Methyl orange (MO) is an anionic, acidic, and water-soluble azo dye widely used in the textile industry [61,62]. It is also released from diverse sources such as research laboratories, food processing, paper manufacturing, printing, pharmaceuticals, plastics, leatherworking, cosmetics, and dye production [62–64]. This azo dye’s molecular structure includes aromatic and -N = N- groups, making it highly toxic, carcinogenic, and teratogenic [65–67]. MO poses severe health risks, impacting kidney and liver function, the central nervous system, brain, and reproductive systems [64,68,69].

Although studies on dye adsorption onto MPs are limited, recent investigations have explored various interactions, including malachite green on nylon MPs [70], methylene blue on PE [36], crystal violet on naturally aged MPs [17], rhodamine B on polyvinyl chloride (PVC) MPs [49], and MO with malachite green on polyamide MPs [71]. Yet there are no research has explored the interaction between specific aged polymers and anionic dyes under varying environmental conditions. Notably, the effects of key factors on adsorption and desorption dynamics. Understanding the interactions between MO and environmentally aged MPs (PE, PET, and PS) will shed light on the role of MPs as pollutant carriers and their potential ecological impacts in aquatic environment.

The main objectives of this study are: 1) to identify the role of environmentally aged MPs properties on MO adsorption, 2) to investigate the influence of controlling factors (contact time, pH, salinity, HA, adsorbent, and adsorbate doses) on MO adsorption onto the MPs, 3) to explore the adsorption performance of MO on the MPs in a freshwater environment, and 4) to evaluate the desorption efficiency of MO from MPs in both freshwater and marine environment.

## 2. Materials and methods

### 2.1 Chemicals and reagents

MO was procured from Sigma-Aldrich (MO, USA). Sodium hydroxide (NaOH) and sodium chloride (NaCl) were obtained from Merck Specialities Private Limited (Mumbai, India), while hydrochloric acid (HCl) was sourced from Merck (Darmstadt, Germany). Humic acid (HA) was acquired from Loba Chemie Private Limited (Mumbai, India). A stock solution of MO (1000 mg/L) was prepared using double-distilled water and stored at 4°C until use. All working standards were freshly prepared from the stock solution through dilution on the day of the experiments.

### 2.2 Preparation and characterization of adsorbent

Environmentally aged PE, PET, and PS plastics were collected from a waste dumping site located in Jashore City, Bangladesh. They were then ultrasonically washed (GT SONIC, VGT-2227QTD, Germany) with distilled water to remove adhering particles from the plastic surfaces and dried at 70°C in an oven (Labtech LDO–150F, Korea). The dried PE, PET, and PS plastics were then cut into small pieces (< 5 mm in length) and sieved to obtain a size range between 0.5 mm and 1 mm. Finally, the MPs were stored in a borosilicate glass bottle and kept in desiccators for further studies.

FTIR spectra of the prepared MPs were recorded before and after adsorption of MO in an FTIR spectrometer (NICOLET IS20, Thermo Scientific, USA) in the range of 500–4,000 cm^-1^ with 16 co-scans collected at 4 cm^-1^ resolutions, and well-equipped with attenuated total reflection (ATR) units. The obtained spectra were processed with Omnic software (OMNIC 8.2) from Nicolet Instrument Corp. (Madison, WI, USA) and identified by comparing them with the internal polymer spectra library databases (Hummel polymer sample library, HR Nicolet Sampler Library, Aldrich condensed Phase Sample Library, etc). Finally, the spectrum obtained by ATR-FTIR that matched >70% to the reference database to confirm the specific kind of polymer. The surface morphology of the MPs was observed before and after the adsorption of MO using a field emission scanning electron microscope (FE–SEM, Zeiss Sigma, Carl Zeiss, Germany) with an accelerating voltage of 10 kV. Before imaging, the samples were coated with a thin gold layer using an ion sputtering device (Hitachi E-1045, Japan) to improve image quality and avoid the buildup of local electrical charges. The pH of the zero-point charge (pH*zpc*) of the MPs was obtained by adding 1 g/L of MPs to 100 mL of 0.01 M NaCl solution with different pH values (pH 2–11) in Erlenmeyer flasks at room temperature for 48-hour reaction periods [72]. The final pH of each solution was determined. The difference between the final and initial pH (ΔpH) values was then plotted against the initial pH values, and the point where ΔpH is zero was taken as the pH*zpc*.

### 2.3 Adsorption experiments

All experiments were performed using 50 mL of working solution in 60 mL glass screw-cap tubes at room temperature (25 ± 2°C). MO adsorption onto MPs was conducted through batch experiments using a suspension mixer (SM-3000, Digisystem Laboratory Instruments Inc., Taiwan) at 120 rpm. Experimental conditions included varying contact times (0.5–96 hours), pH levels (pH 2–11), adsorbent doses (1–15 g/L), initial MO concentrations (5–50 mg/L), sodium chloride (NaCl) concentrations (0–16 g/L), and humic acid (HA) concentrations (0–50 mg/L) in a freshwater environment. To investigate the influence of pH on adsorption, the solution pH was adjusted using 0.1 N HCl or 0.1 N NaOH. After completing each experiment, samples were filtered using glass microfiber filter paper (GF/B, Whatman, USA) to remove adsorbent particles. The MO concentration in the filtrate was measured using a UV-visible spectrophotometer (HACH DR 3900, USA) at a wavelength of 464 nm. Duplicate experiments were conducted, and mean values were used for analysis. The amount of MO adsorption at equilibrium and the removal (R (%)) were calculated by using equations (1) and (2), respectively.


$$q_{e}=\;\frac{{(C}_{0}\;-C_{e})V}{m_{s}}\mathrm{\backslash ]}$$ (1)

$$R\;(\mathrm{\backslash nonumber}\;\%)=\;\frac{\left(C_{0}\;-\;C_{e}\right)}{C_{0}}\times 100\mathrm{\backslash ]}$$ (2)

Where C_o_ and C_e_ are the initial and equilibrium MO concentrations (mg/L), respectively. *q*_*e*_ is the equilibrium MO adsorption capacity (mg/g). V is the volume of solution (L) and m_s_ is the mass of the adsorbent (g).

Adsorption kinetics experiments were conducted by adding 1 g/L of adsorbent to 50 mL of MO solution (10 mg/L concentration) at pH 7, with stirring at 120 rpm at room temperature (25 ± 2°C). Samples were collected at specified time intervals (0.5, 1, 2, 4, 8, 12, 16, 24, and 48 hours), filtered, and analyzed.

Adsorption isotherm experiments were carried out using 50 mL of MO solutions with varying initial concentrations (5, 10, 20, 30, 40, and 50 mg/L). A fixed adsorbent dose of 1 g/L was added to each tube, with solution pH maintained at 7 and room temperature (25 ± 2 °C) for equilibrium contact time.

### 2.4 Box-Behnken response surface methodology (BB RSM)

A three-factor, three-level Box-Behnken experimental design comprising 17 tests was employed using Stat-Ease software (Design-Expert 13.0, Stat-Ease, Inc., Minneapolis, USA) in combination with response surface modeling (RSM). This approach was used to identify the optimal conditions for maximizing the adsorption ofMO) dye onto MPs. The response variable was the MO removal efficiency, while the experimental factors—denoted as A, B, and C—represented the coded terms for the three independent test variables: pH, initial MO dye concentration, and MPs dose, respectively (S1 Table). The experimental design levels were set at −1, 0, and +1 (S1 Table). The RSM polynomial equation (Equation 3) was utilized to model and predict MO dye removal efficiency from the aqueous solution:


$$Y=\beta_{0\;}+\sum_{i=1}^{n}\beta_{i}X_{i}+\sum_{i=n}^{n}\beta_{ij}X_{i}X_{j}+\sum_{i=1}^{n}\beta_{ii}X_{i}^{2}+\varepsilon \mathrm{\backslash ]}$$ (3)

Where, Y is the predicted response (MO removal efficiency), β_0_ and β_i_ are constant and linear coefficients, respectively. β_ij_ is the interaction coefficients, β_ii_ is the quadratic coefficients, X_i_ and X_j_ are the coded values of the process variables, ε is the error, and n is the number of variable studies.

### 2.5 Desorption experiments

The saturated adsorbent MPs were collected after the adsorption process reached equilibrium and subsequently dried in a drying oven at 60°C. For the desorption study, 50 mg of dried MO-loaded MPs were combined with 50 mL of freshwater and simulated seawater (details provided in the supplementary materials) at the natural pH levels of the solutions. The mixtures were agitated at ambient temperature (25 ± 2°C) and a stirring speed of 120 rpm for 48 hours using a suspension mixer. The desorption efficiency (D %) was estimated with the equation (4).


$$D\mathrm{\backslash nonumber}\;\%=\frac{C_{d}\;V_{d}}{{(C}_{o}-C_{e})\;V_{i}}\times 100\mathrm{\backslash ]}$$ (4)

Where C_o_ (mg/L) is the initial dye concentration and C_e_ (mg/L) is the dye concentration at equilibrium, C_d_ (mg/L) indicates the desorbed dye concentration in the desorption process. The volume of fresh or simulated seawater in the desorption study is denoted by V_d_ (L), and dye solution as V_i_ (L)_._

### 2.6 Data analysis and model fitting

To analyze the kinetic behavior of the adsorption process, Lagergren’s pseudo-first-order model [73] and Ho’s pseudo-second-order model [74] were utilized. Additionally, the diffusion mechanism and the potential rate-controlling steps were examined using the inter-particle diffusion model [75]. To further understand the interaction between the adsorbate and adsorbent, and to evaluate the nature of these interactions, two equilibrium adsorption isotherm models—Langmuir [76] and Freundlich [77]—were applied. The respective linear equations for these models are presented in Table in S2 Table.

### 2.7 Quality control and quality assurance

Environmentally aged PE, PET, and PS plastics were collected and prepared for batch adsorption process by soaking with de-ionized water then use ultra-sonication to remove contaminants from plastics cleaning followed by thoroughly washing with distilled water. Plastics chopped into small pieces using scissors and then dried for one days at 70°C. After that, by grinding and sifting, the PE, PET, and PS MPs particle were store in vacuum desiccator for future studies.

All the prepared solutions (stock solution and the working solution) for adsorption process were prepared with de-ionized water and the amounts of solute were weight accurately with digital weight machine. Each batch experiment was carried out in a glass screw-cap tube with a 60 mL volume capacity. Glassware and other experimental materials were cleaned and dried before use. Each clean glass tube was first filled with a specific amount of MPs, and then a layer of Teflon tape was created to cover the glass tube. Then, using a syringe, prepared working solutions of MO dye were injected into each tube. After vertical shaking, the solution was filtered by GF/ B Whatman 1 mm filters with a diameter of 47 mm was used to remove the adsorbent after each experiment to filter the samples at a specific volume. Then, absorbance was measured using a UV visible spectrometer at a particular wavelength.

To minimize the risk of contamination during sample processing, all experimental work was conducted in a clean enclosed chamber. Throughout the procedures laboratory personnel were cotton lab coats and latex gloves to further reduce external contamination sources. To ensure the accuracy of the adsorption data, blank control samples were prepared and processed during the exact same procedures as the test samples. Furthermore, all adsorption experiments were performed in triplicate under the same experimental conditions to verify reproducibility. The consistencies observed across these replicates confirm the reliability and repeatability of the results.

## 3. Results and discussion

### 3.1 Identification and characteristics of MPs

**Fig 1:**
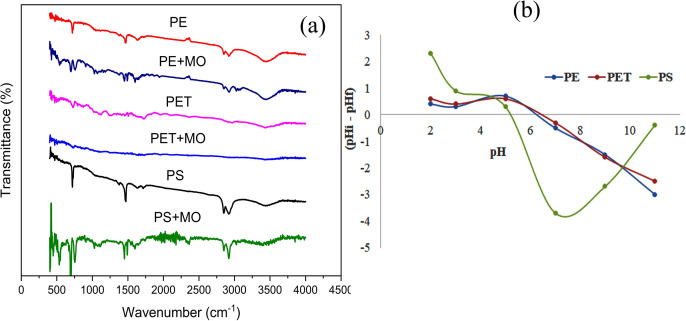
(a) FTIR spectra of the MPs: before (PE, PET and PS) and after (PE + MO, PT + MO and PS + MO) adsorption of MO; and (b) pH of point zero charges of PE, PET and PS MPs.

Fig 1(a) illustrates the FTIR results before and after the adsorption of MO onto PE, PET, and PS. In the FTIR spectrum of PE, a strong peak at 3434 cm ⁻ ¹ corresponds to O–H vibrations, while bands at 2919 cm ⁻ ¹ and 2850 cm ⁻ ¹ are associated with the bending vibration of C–H. Other notable peaks include a C = O vibration at 1715 cm ⁻ ¹, an aromatic ring vibration at 1471 cm ⁻ ¹, and a pronounced peak around 719 cm ⁻ ¹, attributed to the stretching vibration of C–Cl [78]. For PS, a peak at 3391 cm ⁻ ¹ corresponds to O–H stretching vibration, while CH₂ molecular vibrations are observed at 3026 cm ⁻ ¹, 2920 cm ⁻ ¹, and 2851 cm ⁻ ¹. Additionally, peaks related to the aromatic ring vibrations appear at 1600 cm ⁻ ¹, 1491 cm ⁻ ¹, 1028 cm ⁻ ¹, and 753 cm ⁻ ¹ [79]. In the FTIR spectrum of PET, O–H stretching is observed at 3429 cm ⁻ ¹, CH₂ wagging at 2969 cm ⁻ ¹, O = H stretching at 1716 cm ⁻ ¹, C–O rocking at 1264 cm ⁻ ¹, and benzene ring stretching at 873 cm ⁻ ¹ and 732 cm ⁻ ¹ [80]. These findings indicate that the MPs possess surface functional groups capable of linking and interacting with MO molecules (S3 Table).

SEM images of the MPs reveal distinctly irregular profiles and relatively wrinkled surfaces, offering a large surface area for interaction with MO dye molecules (S1(a), S1(c) and S1(e) Fig). The study indicates that the surfaces of the MPs were moderately saturated with MO dye molecules following adsorption (S1(b), S1(d) and S1(f) Fig).

The ΔpH vs. initial pH curve (Fig 1(b)) reveals the pHzpc values of the MPs to be 5.2, 6.7, and 6.8 for PS, PE, and PET, respectively. These pHzpc values suggest that the MPs exhibit a positively charged surface when the pH is below the pHzpc and a negatively charged surface when the pH exceeds the pHzpc.

### 3.2 Effects of the different controlling factors on MO adsorption on MPs

#### 3.2.1 Effect of contact time.

**Fig 2:**
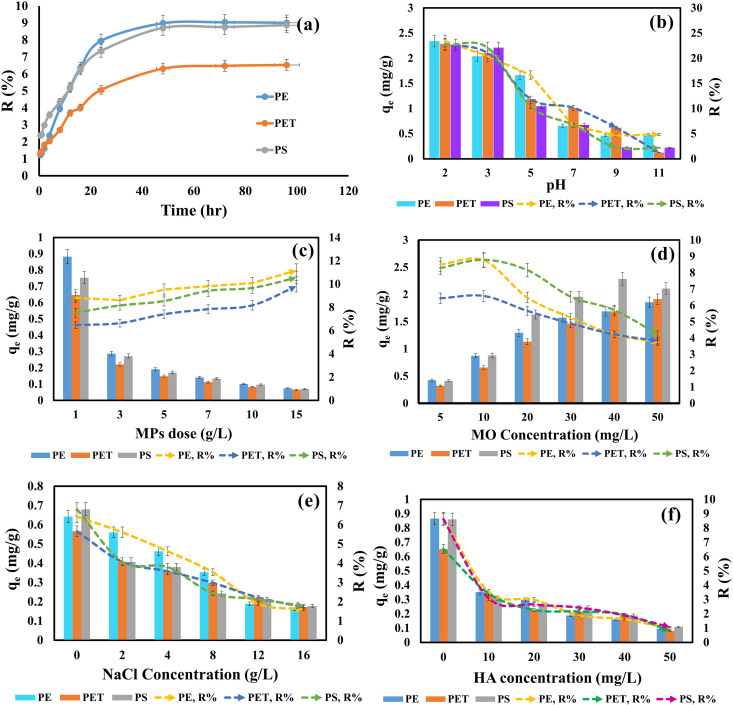
Effects of different controlling factor on MO adsorption by the MPs: (a) Contact time, (b) pH, (c) MPs doses, (d) Initial concentration, (e) NaCl concentration, and (f) HA concentration.

To evaluate the effect of contact time on MO adsorption by MPs, experiments were conducted with varying contact times (0.5–96 hr) under the following conditions: an initial MO dye concentration of 10 mg/L, an adsorbent dose of 1 g/L, pH 7, a stirring speed of 120 rpm, and a temperature of 25 ± 2°C. The results (Fig 2(a)) revealed three distinct phases in the adsorption process. During the initial phase (0.5–2 hr), the adsorption rate was rapid due to the high MO concentration and the abundance of active sites on the adsorbent surface. In the second phase (2–48 hr), the rate of adsorption decreased as the availability of active sites diminished. Finally, adsorption equilibrium was achieved at 48 hr, which was subsequently chosen as the optimal contact time for further experiments.

Under these experimental conditions, MO removal efficiencies were recorded as 9.0% for PE, 6.3% for PET, and 8.7% for PS. Consistent with these findings, recent studies report that dye adsorption onto MPs typically reaches equilibrium within 24–72 hr, depending on the dye and plastic types.[17,28,36,49,81].

#### 3.2.2 Effect of pH.

The adsorption of MO onto MPs across varying pH levels (pH 2–11) is depicted in Fig 2(b), demonstrating that MO adsorption is strongly influenced by the solution pH. Removal efficiency values for PE (23.40–4.83%), PET (22.80–1.23%), and PS (22.64–2.21%), as well as adsorption capacities for PE (2.34–0.48 mg/g), PET (2.28–0.12 mg/g), and PS (2.26–0.22 mg/g), consistently decreased as pH increased (pH 2–11).

Maximum removal efficiency was observed at lower pH levels (Fig 2(b)), attributed to electrostatic attraction between anionic MO dye molecules and positively charged adsorbent surfaces. Electrostatic sorption occurs when a negatively charged dye molecule (like methyl Orange) interact with a positively charged surface site on MPs [82]. Puckowski et al. reported that acidic environments significantly enhance pollutant adsorption onto MPs [83]. In contrast, as pH rises, MO removal efficiency and adsorption capacity progressively decline (Fig 2(b)) due to electrostatic repulsion between anionic MO dye molecules and negatively charged adsorbent surfaces (Fig 1(b)) [68,84].

#### 3.2.3 Effect of adsorbent dose.

The effect of MPs dose on MO adsorption is illustrated in Fig 2(c), indicating that MO removal efficiency increased with higher MPs doses (PE: 8.82–11.17%; PET: 6.47–9.80%; PS: 7.53–10.55%) as the dose increased from 1 to 15 g/L. This improvement in removal efficiency can be attributed to the larger surface area and the greater availability of active sites on the adsorbent surface [52].

However, a higher adsorbent dose (1–15 g/L) resulted in a reduced adsorption capacity (PE: 0.88–0.07 mg/g; PET: 0.64–0.06 mg/g; PS: 0.75–0.07 mg/g), likely due to competition or overlapping of MO molecules on the MPs, such as molecular aggregation. Adsorption of organic and inorganic ions is known to depend on the adsorbent dose, with adsorption amounts decreasing as the adsorbent dose increases [17,37,85].

#### 3.2.4 Effect of initial MO dye concentration.

The removal rate of MO decreases with increasing initial MO concentrations, as shown in Fig 2(d). Removal efficiencies for PE (8.76–3.71%), PET (6.57–3.82%), and PS (8.79–4.22%) decline as the initial concentration of MO rises. While the adsorption capacity of MPs remains constant at a fixed adsorbent dose, removal efficiency is significantly influenced by the initial concentration of MO dye in the solution. At higher MO concentrations, the saturation of active sites on the adsorbent surface leads to excess MO dye remaining unadsorbed in the solution.

The adsorption capacities of MPs (PE: 0.87–1.85 mg/g; PET: 0.65–1.91 mg/g; PS: 0.87–2.11 mg/g) increase with rising MO dye concentrations from 5 to 50 mg/L at a fixed MP dose of 1 g/L (Fig 2(d)). This trend is attributed to the greater driving force that enhances the transfer of MO dye from the liquid phase to the solid phase in aqueous solutions.

#### 3.2.5 Effect of salinity.

NaCl, abundantly present in oceans, rivers, and lakes, plays a critical role in the adsorption of pollutants onto MPs [80]. Fig 2(e) illustrates the effect of salinity on MO adsorption by MPs, showing a gradual decrease in adsorption efficiency with increasing NaCl concentration (0–16 g/L). The removal efficiencies for PE (6.42–1.61%), PET (5.65–1.72%), and PS (6.80–1.78%) declined as NaCl concentration increased, primarily due to heightened competition between excess Na⁺ ions and MO dye molecules for adsorption sites on the MPs surface [86].

Moreover, the presence of Na⁺ ions reduces electrostatic interactions between MPs and MO by neutralizing the negative charges on the active adsorption sites of MPs [87]. Additionally, the increased cohesive energy caused by Cl⁻ ions enhances the cohesive density of MP chains, reducing the availability of free sites on the MPs and thereby diminishing the adsorption rate [88].

Electrostatic interaction is shown to be a crucial factor in the adsorption of MO onto MPs. Consistent with these findings, studies by Chen et al., Du et al., You et al., Zhong et al., and Xu have demonstrated that salinity reduces the adsorption of pollutants by MPs [28,36,49,89,90] a trend also observed in this study.

#### 3.2.6 Effect of humic acid.

Humic acid (HA), a prominent component of natural organic matter (NOM), plays a crucial role in the transport, binding, analysis, and remediation of pollutants in natural environments due to its widespread occurrence and chemical reactivity [91]. Fig 2(f) illustrates the effect of HA concentration (0–50 mg/L) on MO dye adsorption onto MPs. A significant decline in MO adsorption was observed with increasing HA concentrations, with removal efficiencies decreasing for PE (8.66–0.96%), PET (6.53–0.79%), and PS (8.60–1.07%).

Previous research has consistently demonstrated the inhibitory effects of HA on pollutant adsorption onto MPs [46,90,92,93]. As a macromolecular organic substance, HA can alter or block the surface of MPs, forming a barrier that restricts the adsorption of MO ions at active sites [94,95].

#### 3.2.7 Effect of the natural environment.

The objective of this experiment was to investigate the actual adsorption behavior of MO dye onto MPs in a natural freshwater environment, using Padma River water as a representative sample. The results (S2 Fig) revealed that the adsorption of MO onto MPs followed the order: PS > PET > PE. Adsorption capacities in the natural environment were recorded as PE (0.55–1.91 mg/g), PET (0.62–1.96 mg/g), and PS (0.73–2.13 mg/g), which were similar to those observed in controlled adsorption studies (PE: 0.87–1.85 mg/g; PET: 0.65–1.91 mg/g; PS: 0.87–2.11 mg/g) illustrated in Fig 2(d).

### 3.3 Adsorption kinetics

**Fig 3:**
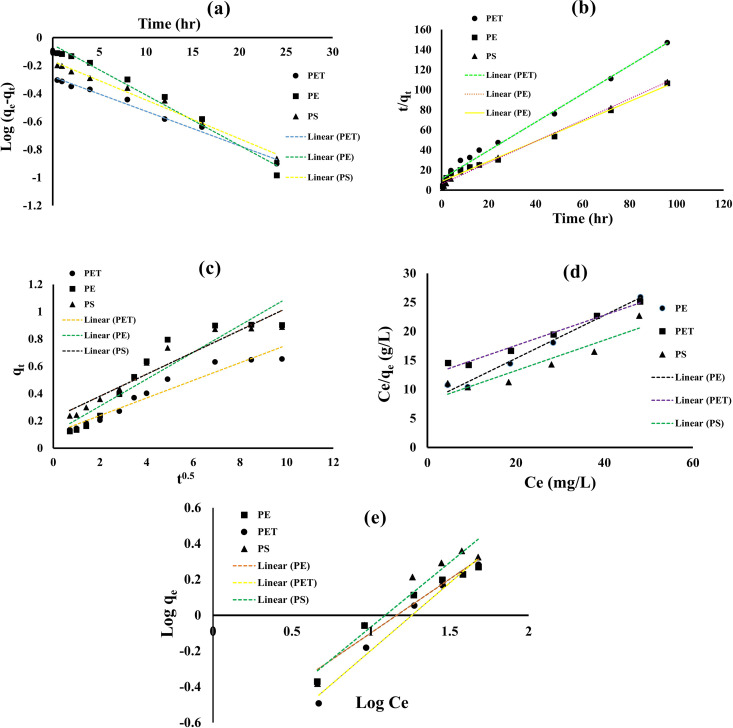
(a) Pseudo first order, (b) Pseudo second order, (c) Intra-particle diffusion kinetics Models, (d) Langmuir, and (e) Freundlich Isotherm models for MO adsorption on the MPs (PE, PET, and PS).

In this study, the adsorption behavior of MO onto MPs was analyzed using Lagergren’s pseudo-first-order, Ho’s pseudo-second-order, and intraparticle diffusion models (Fig 3). Experimental data were compared with the predictions of these adsorption kinetics models based on the correlation coefficient (R²), as summarized in Table in S4 Table. The pseudo-second-order kinetic model yielded higher R² values for MO adsorption compared to the pseudo-first-order model, indicating a better fit. The calculated adsorption capacities derived from the pseudo-second-order model (qe,cal: 1.003 mg/g for PE, 0.708 mg/g for PET, and 0.942 mg/g for PS) closely aligned with the experimental values (qe,exp: 0.878 mg/g for PE, 0.642 mg/g for PET, and 0.871 mg/g for PS) presented in S4 Table suggesting that chemisorption which indicates the chemical bonds between adsorbate molecules and the adsorbents [82] is the primary mechanism controlling the adsorption process [96,97]. The performance of these MPs compare with other adsorbents reported in Table in S5 Table.

Diffusion mechanisms were further investigated using the intraparticle diffusion model. The intraparticle diffusion plot did not pass through the origin (Fig 3(c)), indicating that external diffusion, involving the aggregation of MO molecules on the adsorbent surface, was the rate-limiting step for MO adsorption onto MPs. Although internal diffusion, referring to the transport of MO molecules within the adsorbent particles, may also occur, it was not identified as the rate-limiting step in this study [28].

These results are consistent with findings from prior research, including studies on methylene blue dye adsorption on aged and virgin PE-MPs [36], crystal violet adsorption onto aged polyethylene and polypropylene MPs [17], and the adsorption of malachite green [70] and Rhodamine B [49] dyes onto MPs.

### 3.4 Adsorption isotherms

The equilibrium experimental data for MO adsorption onto MPs were analyzed using the Langmuir and Freundlich isotherm models (Fig 3). The adsorption isotherms and fitting results are summarized in Table in S6 Table. The findings indicate that the Langmuir isotherm provided a better fit for the adsorption of MO onto PE and PET, whereas the Freundlich isotherm better described the adsorption onto PS (S6 Table). Similar trends have been reported for chlorobenzenes and trifluralin adsorption on PE [98], rhodamine B adsorption on PET [49], and polychlorinated biphenyl adsorption on PS [99].

These results suggest that MO molecules form a monolayer with a uniform distribution on PE and PET surfaces. In contrast, the PS surface exhibits non-uniformity, with heterogeneous sites for MO adsorption. PS structure consists of a long hydrocarbon chain with a phenyl group gives PS unique properties allow interaction with other material [100]. PS holds aromatic structure with benzene rings that promote π-π interaction with dye molecules, contributing to heterogeneous adsorption sites. Additionally environmental aging can introduce oxygen containing functional groups and surface irregularities enhancing surface heterogeneity [101–103]. The Langmuir isotherm determined the maximum monolayer adsorption capacities for MO as 2.86 mg/g for PE, 3.64 mg/g for PET, and 3.81 mg/g for PS (S6 Table).

The RL values derived from the Langmuir isotherm (PE: 0.29–0.81, PET: 0.48–0.90, PS: 0.31–0.82) were between 0 and 1, confirming that MO adsorption onto MPs was favorable under the studied conditions. Similarly, the adsorption intensity (n) values (PE: 1.66, PET: 1.32, PS: 1.38) were greater than 1, and the Freundlich constant (KF) values (PE: 0.19, PET: 0.12, PS: 0.16) further demonstrated that the adsorption process was favorable for all MPs [104].

### 3.5 Box–Behnken experimental design and optimization of process variable by RSM

The data presented in Table in S7 Table lists the pH of the adsorbate, initial MO dye concentration, adsorbent dose, and the responses to the examined reactions. MO removal percentages ranged from 3.71–23.41% for PE, 1.23–22.8% for PET, and 2.21–22.64% for PS MPs. The highest MO removal was observed under acidic conditions (pH 2) with an adsorbent dose of 1 g/L, yielding removal efficiencies of 23.41% for PE, 22.8% for PET, and 22.64% for PS.

These results highlight the influence of solution pH on MO adsorption, indicating that the adsorbent surface acquires a net positive charge under acidic conditions. As pH decreases, the electrostatic interactions between the adsorbent surface and the acidic dye (MO) become more prominent, enhancing adsorption efficiency regression analysis was performed to develop response functions fitted to the experimental data. Equations (5), (6), and (7) represent the roles of specific variables (solution pH, MO dye concentration, and MPs dose) and their interaction effects. The adsorption of MO was significantly impacted by the interplay of these variables, as detailed in Equations (5–7):



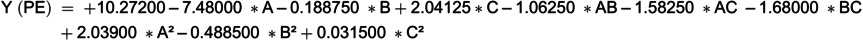

(5)




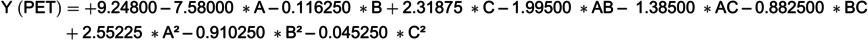

(6)




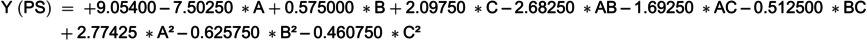

(7)


**Fig 4:**
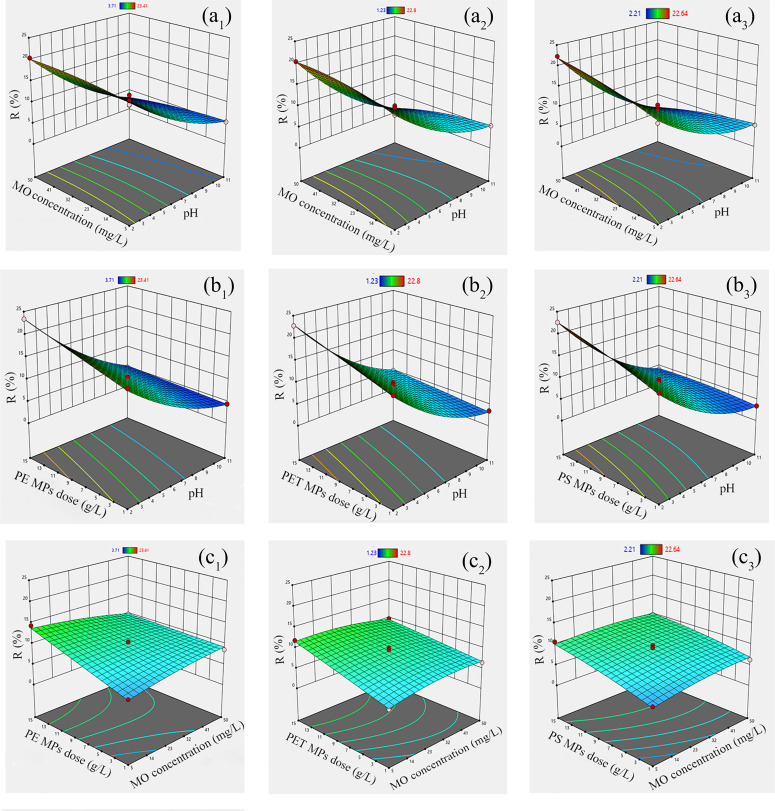
3D response surface model of MO dye removal efficiency: MO concentration (mg/L) and pH (a_1_ to a_3_), MPs dose (g/L) and pH (b_1_ to b_3_), and MPs dose (g/L) and MO concentration (mg/L) (c_1_ to c_3_) by PE, PET and PS, respectively.

The effects of these variables on MO removal efficiency (R%) for PE, PET, and PS MPs are illustrated in Fig 4. At low pH (2), adsorption is enhanced due to strong surface charge interactions, resulting in higher removal efficiencies (Runs 4, 15, 16, 17). At neutral pH (6.5), adsorption is moderate (Runs 1, 2, 5, 6, 8, 13) as electrostatic forces weaken, while at high pH (11), adsorption decreases significantly (Runs 3, 7, 9, 10, 11) due to repulsion effects.

The interaction between pH and MO concentration further reveals adsorption efficiency variations. At low MO concentration (5 mg/L), lower pH improves adsorption slightly (Runs 1, 4, 7, 14), while at medium concentration (27.5 mg/L), acidic conditions consistently enhance adsorption (Runs 2, 5, 6, 8, 9, 13, 17). For high MO concentrations (50 mg/L), low pH (Run 15) results in significantly greater adsorption compared to high pH (Run 3).

Analysis of variance (ANOVA) was conducted to assess the significance of the second-order model (S8 Table). ANOVA results indicate that solution pH has a negative impact on MO adsorption, as MO is an anionic dye, and electrostatic attraction occurs between the positively charged MPs surface and MO dye molecules in an acidic environment. The model F-values (PE: 142.91, PET: 162.31, PS: 98.42) demonstrate that the model terms are statistically significant for PE, PET, and PS (P < 0.0001), as shown in Table in S8 Table. The P-value for “lack of fit” for all adsorbents was > 0.01, suggesting that random variations or noise may contribute to the lack of fit F-values without significantly affecting the model’s reliability.

Key model terms based on variable interactions were identified as follows: PE = A, C, AB, AC, BC, A²; PET = A, C, AB, AC, BC, A², B²; and PS = A, C, AB, AC, A². Solution pH and adsorbent dose are critical factors influencing MO adsorption, as outlined in Table in S7 Table. The coefficients of determination (R² values) for PE (0.994), PET (0.995), and PS (0.992) and adjusted R² values (PE: 0.987; PET: 0.989; PS: 0.982) indicate that the model accurately explains 99% of the response variability.

**Fig 5:**
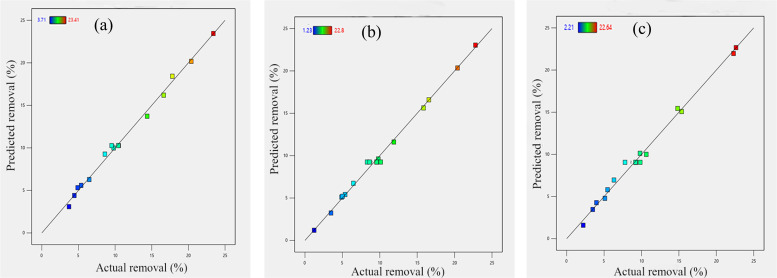
Predicted removal efficiency versus actual removal efficiency of MO by (a) PE, (b) PET, and (c) PS MPs.

As illustrated in Fig 5, the experimental data points closely align with the model’s predictions, demonstrating a strong fit. The agreement between R² and adjusted R² values further confirms the model’s robustness. Additionally, the Adeq Precision values (signal-to-noise ratio) of PE (41.53), PET (46.01), and PS (34.44) exceed the desired threshold of 4, signifying sufficient signal strength. This model is suitable for navigating the design space.

### 3.6 Desorption behavior of MPs in fresh and simulated seawater

**Fig 6:**
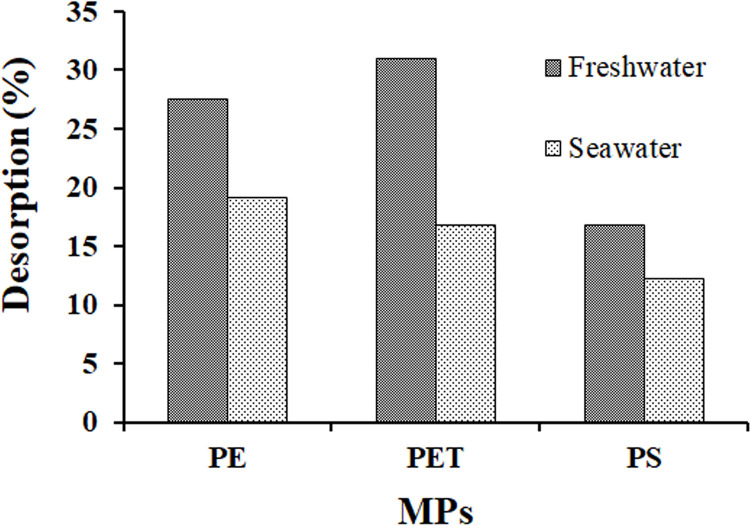
Desorption of MO from (a) PE, (b) PET, and (c) PS MPs under freshwater and simulated seawater environment.

This study also explores the desorption behavior of MPs in freshwater and simulated seawater environments, as illustrated in Fig 6. During the desorption phase, the desorption rates of MO were observed to be higher in freshwater (PE: 27.48% (2.83 mg/g), PET: 30.93% (4.5 mg/g), PS: 16.79% (2.5 mg/g)) compared to seawater (PE: 19.11% (2.76 mg/g), PET: 16.75% (2.5 mg/g), PS: 12.18% (1.93 mg/g)).

This difference suggests that NaCl molecules in seawater compete with MO molecules for adsorption sites on the MPs, thereby influencing the balance between adsorption (the binding of MO molecules to the MPs surface) and desorption (the release of MO molecules from the MPs surface) [80]. Such processes highlight the regulatory role of NaCl in controlling adsorption and desorption mechanisms.

Consequently, MPs that have adhered to pollutants may pose greater risks to freshwater ecosystems and their associated organisms, as higher desorption rates in freshwater can enhance the mobility and bioavailability of pollutants, exacerbating their environmental impact.

## 4. Conclusions

This study assesses the adsorption and desorption capacity of environmentally aged PE, PET, and PS MPs for MO dye using batch experiments. Solution pH negatively impacts MO adsorption, while high concentrations of Na⁺ and HA obstruct the process. PE and PET adsorb MO through monolayer chemisorption, while PS follows multi-layer chemisorption. Adsorption aligns with the pseudo-second-order kinetic model and fits well with Langmuir and Freundlich isotherms. PS achieves the highest adsorption capacity (3.80 mg/g), followed by PET (3.64 mg/g) and PE (2.68 mg/g).

RSM results show significant agreement between experimental and predicted data, highlighting the role of pH, dye concentration, and MPs dose in MO removal. Electrostatic interactions, hydrogen bonding, and hydrophobic interactions are key factors driving adsorption. Interestingly, MO exhibited greater desorption rates in freshwater compared to seawater.

Finally, MPs serve as pathways for MO dye migration into ecosystems via adsorption and desorption. Further studies are needed to explore MPs’ interactions with pollutants and evaluate dye release dynamics in real-world environments.

### 4.1 Limitations and future research direction

This study provides valuable insights into the adsorption and desorption behavior of methyl orange dye on PE, PET, and PS MPs under varying environmental conditions. However, there are certain limitations such as the long term stability and reusability of the MPs as adsorbents were not assessed through cyclic sorption experiment, secondly, while seawater conditions were simulated using NaCl to represent ionic strength, real sea water contains a more complex mixture of ions which may influence sorption dynamics differently. Additionally quantitative surface characterization such as BET surface area and porosity analysis was not performed which could have provided a deeper understanding of the adsorption properties. Future research should incorporate repeated adsorption –desorption to evaluate long term stability of adsorbents and explore the effects of a variety of ionic matrix in natural sea water to improve the environmental relevance and predictive accuracy.

## Supporting information

S1 Table Independent variables, their experimental range and three levels of these variables. (DOCX)

S2 Table The linear equations used in kinetics and isotherm models. (DOCX)

S3 Table Major peaks with functional group of MPs after adsorption. (DOCX)

S4 Table Pseudo-First, Second-Order and Intra-particle diffusion model parameter of MO Adsorption on MPs. (DOCX)

S5 Table Adsorption efficiency of adsorbents (PE, PET and PS MPs) compare with other adsorbents. (DOCX)

S6 Table Langmuir and Freundlich Isotherms model parameters for MO adsorption on MPs. (DOCX)

S7 Table Box–Behnken design matrix for MO dye removal by PE, PET and PS MPs. (DOCX)

S8 Table Analysis of variance (ANOVA) for the response surface quadratic model. (DOCX)

S1 Fig SEM images before MO adsorption and after MO adsorption. (DOCX)

S2 Fig MO dye adsorption on the MPs in freshwater environment. (DOCX)

S1 Data MO dye adsorption and desorption on MPs. (XLSX)
