# Supplementary material for: Adsorption and desorption of methyl orange dye on environmentally aged polyethylene, polyethylene terephthalate and polystyrene microplastics in aquatic environment
Source: PLoS One. 2025 Jul 28;20(7):e0323516. doi: 10.1371/journal.pone.0323516 (PMC12303273; doi:10.1371/journal.pone.0323516)
Supplement: S1 Table — (DOCX) [file pone.0323516.s001.docx]

**S1 Table.** Independent variables, their experimental range and three levels of these variables

| **Variables** | **Factors** | **Coded factors Level** | | |
| --- | --- | --- | --- | --- |
|  |  | **-1** | **0** | **+1** |
| Solution pH | A | 2 | 6.5 | 11 |
| Dye concentration (mg/L) | B | 5 | 27.5 | 50 |
| MPs dose (g/L) | C | 1 | 8 | 15 |
