# Supplementary material for: Adsorption and desorption of methyl orange dye on environmentally aged polyethylene, polyethylene terephthalate and polystyrene microplastics in aquatic environment
Source: PLoS One. 2025 Jul 28;20(7):e0323516. doi: 10.1371/journal.pone.0323516 (PMC12303273; doi:10.1371/journal.pone.0323516)
Supplement: S2 Table — (DOCX) [file pone.0323516.s002.docx]

**S2 Table.** The linear equations used in kinetics and isotherm models.

| **Experiment type** | **Models** | **Equations** |
| --- | --- | --- |
| Kinetics | Pseudo-first-order (PFO) | $\log\left( qe-qt \right)= log\left( qe \right)- \frac{K_{1}}{2.303} t$ |
|  | Pseudo-second-order (PSO) | $t/{q_{t}}=t/{q_{t}}+1/{k_{2}q_{e}^{2}}$ |
|  | The intraparticle diffusion | $q_{t}={K_{diff.}t}^{0.5}+C$ |
| Isotherm | Langmuir | $\frac{C_{e}}{q_{e}}=\frac{1}{q_{max}b} +\frac{c_{e}}{q_{max}}$ |
|  |  | $R_{L}=\frac{1}{1+bC_{0}}$ |
|  | Freundlich | $logq_{e}=\log k_{f}+\frac{1}{n} log C_{e}$ |

Where K_1_ is the pseudo-first-order rate constant (hr^-1^), q_e_, and q_t_ are the amount of MO adsorbed (mg/g) at equilibrium and time, t (hr). K_2_ is the pseudo-second-order rate constant (g/mg/hr^-1^). Where h is the initial adsorption rate (mg/g/hr). K*_diff_*. is the intraparticle diffusion rate constant (mg/g. min^0.5^), which represents the thickness of the boundary layer. C_e_ is the equilibrium concentration (mg/L), q_max_ is the amount of MO at complete monolayer coverage (mg/g), and b (L/mg) is Langmuir constant. The essential feature of the Langmuir isotherm can be expressed in terms of the dimensionless constant separation factor (R_L_) that is used to predict whether an adsorption system is "favourable" or "unfavourable". The adsorption process as a function of R_L_ may be described as R_L_> 1; unfavorable, R_L_ = 1; Linear, 0 <R_L_< 1; favorable and R_L_ = 0; irreversible. Where, K_F_ and n are Freundlich constant and adsorption intensity, respectively.
