# Supplementary material for: Adsorption and desorption of methyl orange dye on environmentally aged polyethylene, polyethylene terephthalate and polystyrene microplastics in aquatic environment
Source: PLoS One. 2025 Jul 28;20(7):e0323516. doi: 10.1371/journal.pone.0323516 (PMC12303273; doi:10.1371/journal.pone.0323516)
Supplement: S3 Table — (DOCX) [file pone.0323516.s003.docx]

**S3 Table:** Major peaks with functional group of MPs after adsorption.

| MPs | Peaks at (cm^-1^) | Band Type | Functional Group |
| --- | --- | --- | --- |
| PE | 3434 | Vibration | O-H |
|  | 2910 | bending vibration | CH_2_ |
|  | 1471 | bending vibration | CH_2_ |
|  | 1715 | aromatic ring vibration | C=O |
|  | 719 | stretching vibration | C=Cl |
|  | 1630 | Vibration | C=N |
| PET | 3429 | stretching | O-H |
|  | 2969 | rocking | CH_2_ |
|  | 1716 | bending vibration | O-H |
|  | 1264 | aromatic ring vibration | C=O |
| PS | 3391 | stretching vibration | O-H |
|  | 3026 | molecular vibration | CH_2_ |
|  | 1732 | aromatic ring vibration | C=O |
|  | 1557 | aromatic ring vibration | N-O |
