# Supplementary material for: Adsorption and desorption of methyl orange dye on environmentally aged polyethylene, polyethylene terephthalate and polystyrene microplastics in aquatic environment
Source: PLoS One. 2025 Jul 28;20(7):e0323516. doi: 10.1371/journal.pone.0323516 (PMC12303273; doi:10.1371/journal.pone.0323516)
Supplement: S4 Table — (DOCX) [file pone.0323516.s004.docx]

**S4 Table.** Pseudo-First, Second-Order and Intra-particle diffusion model parameter of MO Adsorption on MPs.

| **Model** | **Parameter** | **PE-MPs** | **PET-MPs** | **PS-MPs** |
| --- | --- | --- | --- | --- |
| **Pseudo-first-order kinetics** | ***q_e,exp_(mg/g)*** | 0.878 | 0.642 | 0.871 |
|  | ***q_e,cal_(mg/g)*** | 0.751 | 0.533 | 0.678 |
|  | ***K_1_ (h^-1^)*** | -0.082 | -0.054 | -0.063 |
|  | ***R^2^*** | 0.977 | 0.9884 | 0.986 |
| **Pseudo-second-order kinetics** | ***q_e,exp_(mg/g)*** | 0.898 | 0.642 | 0.871 |
|  | ***q_e,cal_(mg/g)*** | 1.003 | 0.708 | 0.9423 |
|  | ***K_2_ (g.mg^-1^h^-1^)*** | 0.113 | 0.175 | 0.181 |
|  | ***h*** | 0.114 | 0.088 | 0.160 |
|  | ***R^2^*** | 0.9914 | 0.9887 | 0.993 |
| **Intra-particle**  **Diffusion Model** | C | 0.1106 | 0.1095 | 0.2197 |
|  | K*_diff_* | 0.0988 | 0.0649 | 0.0806 |
|  | *R^2^* | 0.8788 | 0.9395 | 0.9214 |
