# Supplementary material for: Adsorption and desorption of methyl orange dye on environmentally aged polyethylene, polyethylene terephthalate and polystyrene microplastics in aquatic environment
Source: PLoS One. 2025 Jul 28;20(7):e0323516. doi: 10.1371/journal.pone.0323516 (PMC12303273; doi:10.1371/journal.pone.0323516)
Supplement: S5 Table — (DOCX) [file pone.0323516.s005.docx]

**S5 Table.** Adsorption efficiency of adsorbents (PE, PET and PS MPs) compare with other adsorbents.

| Adsorbents  (MPs) | Adsorbates  (Dye) | Adsorption capacity (mg/g) | Optimum dose (mg) | Adsorbates concentration (mg/L) | pH | References |
| --- | --- | --- | --- | --- | --- | --- |
| Aged PE | CV | 1.69 | 50 | 5-30 | 2-10 | [1] |
| aged PP |  | 4.28 | 50 | 5-30 | 2-10 | [1] |
| PE | MG | 4.52 | 60 | 10-50 | 2-10 | [2] |
| PVC |  | 7.68 | 60 | 10-50 | 2-10 | [2] |
| PA6 |  | 3.04 | 50 | 0-30 | 2-10 | [3] |
| PA66 |  | 2.71 | 50 | 0-30 | 2-10 | [3] |
| PA6 | MO | 6.45 | 50 | 0-30 | 2-10 | [3] |
| PA66 |  | 4.16 | 50 | 0-30 | 2-10 | [3] |
| PE |  | 2.86 | 1000 | 5-50 | 2–11 | This study |
| PET |  | 3.64 | 1000 | 5-50 | 2–11 | This study |
| PS |  | 3.81 | 1000 | 5-50 | 2–11 | This study |
| Aged PE | MB | 8.12 | 50 | 5-40 | 3-11 | [4] |
| PE | RhB | 1.27 | 60 | 2-10 | 2-10 | [2] |
| PVC |  | 2.83 | 60 | 2-10 | 2-10 | [2] |
| PS |  | 1.54 | 20 | 10 | 3–9 | [5] |
| PET |  | 1.28 | 20 | 10 | 3–9 | [5] |

PP=Polypropylene, PA6=Polyamide 6, PA66=Polyamide 66, CV =Crystal Violet, MO= Methyl Orange, MG = Malachite Green, MB= Methylene Blue, RhB= Rhodamine B

**References:**

[1] Du H, Ma H, Xing B. Identification of naturally weathering microplastics and their interactions with ion dyes in aquatic environments. Mar Pollut Bull. 2022;174:113186. <https://doi.org/10.1016/j.marpolbul.2021.113186>.

[2] Zhong Y, Wang K, Guo C, Kou Y, Hassan A, Lu Y, et al. Competition adsorption of malachite green and rhodamine B on polyethylene and polyvinyl chloride microplastics in aqueous environment. Water Sci Technol. 2022;86(5):894–908. <https://doi.org/10.2166/wst.2022.252>

[3] Wang, K., Kou, Y., Wang, K., Liang, S., Guo, G., Wang, W., Lu, Y., & Wang, J. (2023). Comparing the adsorption of methyl orange and malachite green on similar yet distinct polyamide microplastics: Uncovering hydrogen bond interactions. Chemosphere, 340, 139806. <https://doi.org/10.1016/j.chemosphere.2023.139806>

[4] You H, Huang B, Cao C, Liu X, Sun X, Xiao L, et al. Adsorption–desorption behavior of methylene blue onto aged polyethylene microplastics in aqueous environments. Mar Pollut Bull. 2021;167:112287. <https://doi.org/10.1016/j.marpolbul.2021.112287>

[5] Du H, Zhang Y, Jiang H, Wang H. Adsorption of rhodamine B on polyvinyl chloride, polystyrene, and polyethylene terephthalate microplastics in aqueous environments. Environ Technol Innov. 2022;27:102495. <https://doi.org/10.1016/j.eti.2022.102495>
