# Supplementary material for: Adsorption and desorption of methyl orange dye on environmentally aged polyethylene, polyethylene terephthalate and polystyrene microplastics in aquatic environment
Source: PLoS One. 2025 Jul 28;20(7):e0323516. doi: 10.1371/journal.pone.0323516 (PMC12303273; doi:10.1371/journal.pone.0323516)
Supplement: S6 Table — (DOCX) [file pone.0323516.s006.docx]

**S6 Table.** Langmuir and Freundlich Isotherms model parameters for MO adsorption on MPs.

|  | **Langmuir isotherm** | | | | **Freundlich isotherm** | | |
| --- | --- | --- | --- | --- | --- | --- | --- |
|  | ***q_max_(mg/g)*** | ***b (L/mg)*** | ***R^2^*** | ***R_L_*** | ***K_F_*** | ***n*** | ***R^2^*** |
| **PE-MPs** | 2.685 | 0.047 | 0.986 | 0.809-0.297 | 0.199 | 1.660 | 0.9518 |
| **PET-MPs** | 3.642 | 0.023 | 0.995 | 0.904-0.486 | 0.120 | 1.362 | 0.9886 |
| **PS-MPs** | 3.806 | 0.329 | 0.877 | 0.820-0.313 | 0.163 | 1.388 | 0.9383 |
