# Supplementary material for: Adsorption and desorption of methyl orange dye on environmentally aged polyethylene, polyethylene terephthalate and polystyrene microplastics in aquatic environment
Source: PLoS One. 2025 Jul 28;20(7):e0323516. doi: 10.1371/journal.pone.0323516 (PMC12303273; doi:10.1371/journal.pone.0323516)
Supplement: S7 Table — (DOCX) [file pone.0323516.s007.docx]

**S7 Table.** Box–Behnken design matrix for MO dye removal by PE, PET and PS MPs.

|  | **Factor 1** | | | **Factor 2** | | | **Factor 3** | | | **Response 1** | | | **Response 2** | | | **Response 3** | | | **Response 4** | | |
| --- | --- | --- | --- | --- | --- | --- | --- | --- | --- | --- | --- | --- | --- | --- | --- | --- | --- | --- | --- | --- | --- |
| Run | A:pH | | | B:MO concentration (mg/L) | | | C: MPs dose (g/L) | | | pH | | | MO Concentration (mg/L) | | | MPs dose  (g/L) | | | MO Removal (%) | | |
|  | PE | PET | PS | PE | PET | PS | PE | PET | PS | PE | PET | PS | PE | PET | PS | PE | PET | PS | PE | PET | PS |
| 1 | 0 | 0 | 0 | -1 | -1 | -1 | 1 | 1 | 1 | 6.5 | 6.5 | 6.5 | 5 | 5 | 5 | 15 | 15 | 15 | 14.38 | 11.88 | 10.64 |
| 2 | 0 | 0 | 0 | 0 | 0 | 0 | 0 | 0 | 0 | 6.5 | 6.5 | 6.5 | 27.5 | 27.5 | 27.5 | 8 | 8 | 8 | 10.45 | 10.13 | 9.83 |
| 3 | 1 | 1 | 1 | 1 | 1 | 1 | 0 | 0 | 0 | 11 | 11 | 11 | 50 | 50 | 50 | 8 | 8 | 8 | 3.71 | 1.23 | 2.21 |
| 4 | -1 | -1 | -1 | -1 | -1 | -1 | 0 | 0 | 0 | 2 | 2 | 2 | 5 | 5 | 5 | 8 | 8 | 8 | 17.81 | 16.56 | 14.83 |
| 5 | 0 | 0 | 0 | 0 | 0 | 0 | 0 | 0 | 0 | 6.5 | 6.5 | 6.5 | 27.5 | 27.5 | 27.5 | 8 | 8 | 8 | 10.44 | 9.58 | 9.21 |
| 6 | 0 | 0 | 0 | 0 | 0 | 0 | 0 | 0 | 0 | 6.5 | 6.5 | 6.5 | 27.5 | 27.5 | 27.5 | 8 | 8 | 8 | 10.45 | 9.58 | 9.22 |
| 7 | 1 | 1 | 1 | -1 | -1 | -1 | 0 | 0 | 0 | 11 | 11 | 11 | 5 | 5 | 5 | 8 | 8 | 8 | 5.37 | 5.37 | 5.46 |
| 8 | 0 | 0 | 0 | 0 | 0 | 0 | 0 | 0 | 0 | 6.5 | 6.5 | 6.5 | 27.5 | 27.5 | 27.5 | 8 | 8 | 8 | 10.49 | 8.31 | 7.8 |
| 9 | 1 | 1 | 1 | 0 | 0 | 0 | -1 | -1 | -1 | 11 | 11 | 11 | 27.5 | 27.5 | 27.5 | 1 | 1 | 1 | 4.44 | 3.48 | 3.48 |
| 10 | 0 | 0 | 0 | 1 | 1 | 1 | -1 | -1 | -1 | 6.5 | 6.5 | 6.5 | 50 | 50 | 50 | 1 | 1 | 1 | 8.61 | 6.47 | 6.32 |
| 11 | 1 | 1 | 1 | 0 | 0 | 0 | 1 | 1 | 1 | 11 | 11 | 11 | 27.5 | 27.5 | 27.5 | 15 | 15 | 15 | 4.89 | 4.89 | 3.98 |
| 12 | 0 | 0 | 0 | 1 | 1 | 1 | 1 | 1 | 1 | 6.5 | 6.5 | 6.5 | 50 | 50 | 50 | 15 | 15 | 15 | 9.8 | 9.8 | 9.8 |
| 13 | 0 | 0 | 0 | 0 | 0 | 0 | 0 | 0 | 0 | 6.5 | 6.5 | 6.5 | 27.5 | 27.5 | 27.5 | 8 | 8 | 8 | 9.53 | 8.64 | 9.21 |
| 14 | 0 | 0 | 0 | -1 | -1 | -1 | -1 | -1 | -1 | 6.5 | 6.5 | 6.5 | 5 | 5 | 5 | 1 | 1 | 1 | 6.47 | 5.02 | 5.11 |
| 15 | -1 | -1 | -1 | 1 | 1 | 1 | 0 | 0 | 0 | 2 | 2 | 2 | 50 | 50 | 50 | 8 | 8 | 8 | 20.4 | 20.4 | 22.31 |
| 16 | -1 | -1 | -1 | 0 | 0 | 0 | 1 | 1 | 1 | 2 | 2 | 2 | 27.5 | 27.5 | 27.5 | 15 | 15 | 15 | 23.41 | 22.8 | 22.64 |
| 17 | -1 | -1 | -1 | 0 | 0 | 0 | -1 | -1 | -1 | 2 | 2 | 2 | 27.5 | 27.5 | 27.5 | 1 | 1 | 1 | 16.63 | 15.85 | 15.37 |
