# Supplementary material for: Adsorption and desorption of methyl orange dye on environmentally aged polyethylene, polyethylene terephthalate and polystyrene microplastics in aquatic environment
Source: PLoS One. 2025 Jul 28;20(7):e0323516. doi: 10.1371/journal.pone.0323516 (PMC12303273; doi:10.1371/journal.pone.0323516)
Supplement: S8 Table — (DOCX) [file pone.0323516.s008.docx]

| **Source** | **Sum of Squares** | | | **Degree of freedom** | | | **Mean Square** | | | **F-value** | | | **p-value** | | | | | |
| --- | --- | --- | --- | --- | --- | --- | --- | --- | --- | --- | --- | --- | --- | --- | --- | --- | --- | --- |
|  | **PE** | **PET** | **PS** | **PE** | **PET** | **PS** | **PE** | **PET** | **PS** | **PE** | **PET** | **PS** | **PE** | | **PET** | | **PS** | |
| **Model** | 525.22 | 559.46 | 563.30 | 9 | 9 | 9 | 58.36 | 62.16 | 62.59 | 142.91 | 162.31 | 98.42 | < 0.0001 | significant | < 0.0001 | significant | < 0.0001 | significant |
| A | 447.60 | 459.65 | 450.30 | 1 | 1 | 1 | 447.60 | 459.65 | 450.30 | 1096.12 | 1200.18 | 708.12 | < 0.0001 |  | < 0.0001 |  | < 0.0001 |  |
| B | 0.2850 | 0.1081 | 2.64 | 1 | 1 | 1 | 0.2850 | 0.1081 | 2.64 | 0.6980 | 0.2823 | 4.16 | 0.4311 |  | 0.6116 |  | 0.0808 |  |
| C | 33.33 | 43.01 | 35.20 | 1 | 1 | 1 | 33.33 | 43.01 | 35.20 | 81.63 | 112.31 | 55.35 | < 0.0001 |  | < 0.0001 |  | 0.0001 |  |
| AB | 4.52 | 15.92 | 28.78 | 1 | 1 | 1 | 4.52 | 15.92 | 28.78 | 11.06 | 41.57 | 45.26 | 0.0127 |  | 0.0004 |  | 0.0003 |  |
| AC | 10.02 | 7.67 | 11.46 | 1 | 1 | 1 | 10.02 | 7.67 | 11.46 | 24.53 | 20.03 | 18.02 | 0.0017 |  | 0.0029 |  | 0.0038 |  |
| BC | 11.29 | 3.12 | 1.05 | 1 | 1 | 1 | 11.29 | 3.12 | 1.05 | 27.65 | 8.13 | 1.65 | 0.0012 |  | 0.0246 |  | 0.2396 |  |
| A² | 17.51 | 27.43 | 32.41 | 1 | 1 | 1 | 17.51 | 27.43 | 32.41 | 42.87 | 71.61 | 50.96 | 0.0003 |  | < 0.0001 |  | 0.0002 |  |
| B² | 1.00 | 3.49 | 1.65 | 1 | 1 | 1 | 1.00 | 3.49 | 1.65 | 2.46 | 9.11 | 2.59 | 0.1607 |  | 0.0194 |  | 0.1514 |  |
| C² | 0.0042 | 0.0086 | 0.8939 | 1 | 1 | 1 | 0.0042 | 0.0086 | 0.8939 | 0.0102 | 0.0225 | 1.41 | 0.9223 |  | 0.8850 |  | 0.2745 |  |
| **Residual** | 2.86 | 2.68 | 4.45 | 7 | 7 | 7 | 0.4084 | 0.3830 | 0.6359 |  |  |  |  |  |  |  |  |  |
| Lack of Fit | 2.17 | 0.4330 | 2.20 | 3 | 3 | 3 | 0.7229 | 0.1443 | 0.7335 | 4.19 | 0.2568 | 1.30 | 0.0999 | not significant | 0.8534 | not significant | 0.3888 | not significant |
| Pure Error | 0.6897 | 2.25 | 2.25 | 4 | 4 | 4 | 0.1724 | 0.5620 | 0.5627 |  |  |  |  |  |  |  |  |  |
| **Cor Total** | 528.08 | 562.14 | 567.76 | 16 | 16 | 16 |  |  |  |  |  |  |  |  |  |  |  |  |
| **R²** | **0.9946** | **0.9952** | **0.9922** |  |  |  |  |  |  |  |  |  |  |  |  |  |  |  |
| **Adjusted R²** | **0.9876** | **0.9891** | **0.9821** |  |  |  |  |  |  |  |  |  |  |  |  |  |  |  |
| **Predicted R²** | **0.9322** | **0.9814** | **0.9318** |  |  |  |  |  |  |  |  |  |  |  |  |  |  |  |
| **Adeq Precision** | **41.5316** | **46.0135** | **34.4461** |  |  |  |  |  |  |  |  |  |  |  |  |  |  |  |

**S8 Table.** Analysis of variance (ANOVA) for the response surface quadratic model.
