# Supplementary figures and images for: Adsorption and desorption of methyl orange dye on environmentally aged polyethylene, polyethylene terephthalate and polystyrene microplastics in aquatic environment
Source: PLoS One. 2025 Jul 28;20(7):e0323516. doi: 10.1371/journal.pone.0323516 (PMC12303273; doi:10.1371/journal.pone.0323516)

**S1 Fig.** SEM images before MO adsorption : PE (a), PET (c), and PS (e) , and after MO adsorption: PE (b), PET (d) and PS (f).


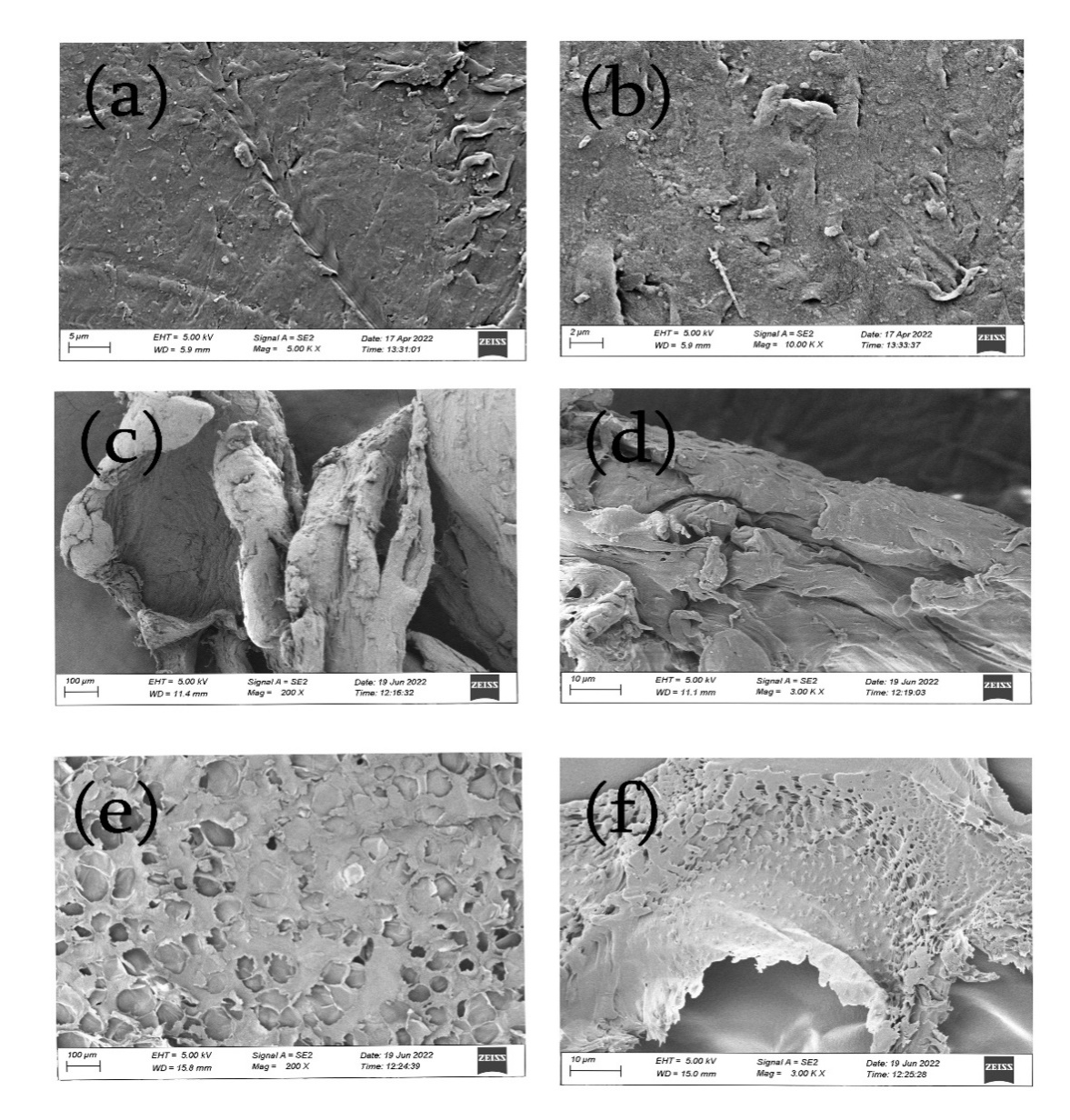

Supplement: S1 Fig — (DOCX) [file pone.0323516.s009.docx]
