# Supplementary material for: Adsorption and desorption of methyl orange dye on environmentally aged polyethylene, polyethylene terephthalate and polystyrene microplastics in aquatic environment
Source: PLoS One. 2025 Jul 28;20(7):e0323516. doi: 10.1371/journal.pone.0323516 (PMC12303273; doi:10.1371/journal.pone.0323516)
Supplement: S2 Fig — (DOCX) [file pone.0323516.s010.docx]

**
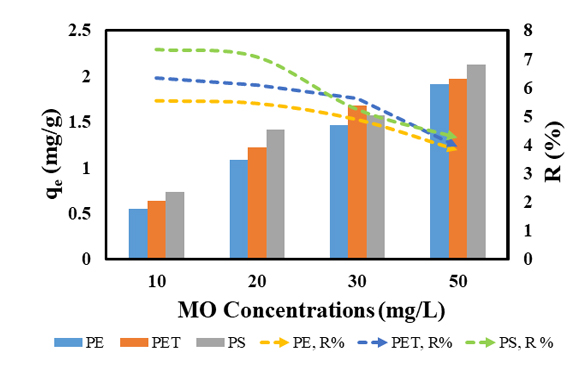
**

**S2 Fig.** MO dye adsorption on the MPs in freshwater environment

**Preparation of freshwater and simulated seawater:**

**Freshwater:** Freshwater was collected from the Padma river, the collected freshwater was filtered through Whatman GF/B filter paper to segregate impurities and unwanted debris. Then the collected freshwater was prepared for the adsorption process. The quality of collected water was found as pH ranges from 7.7-7.9, Electric conductivity (EC) 643 μS/m, Total dissolved solids (TDS) 313 mg/L and salinity was 0.4%.

**Simulated seawater:** To produce simulated seawater a certain amount of NaCl is dissolved in distilled water to prepare a solution with an average salinity of 33.53%.
