# Supplementary material for: Adsorption and desorption of methyl orange dye on environmentally aged polyethylene, polyethylene terephthalate and polystyrene microplastics in aquatic environment
Source: PLoS One. 2025 Jul 28;20(7):e0323516. doi: 10.1371/journal.pone.0323516 (PMC12303273; doi:10.1371/journal.pone.0323516)
Supplement: Attachment — Submitted filename: Response to Reviewers Letter.docx [file pone.0323516.s012.docx]

**Dear Academic Editor,**

Thank you for the opportunity to revise our manuscript (PONE‑D‑25‑18547) titled “Adsorption and desorption of methyl orange dye on environmentally aged polyethylene, polyethylene terephthalate and polystyrene microplastics in aquatic environment.” We appreciate the constructive feedback provided by you and the reviewers. Below we provide a point‐by‐point response to all comments raised.

Reviewer #1: Manuscript Reference: PONE-D-25-18547
Manuscript Title: Adsorption and desorption of methyl orange dye on environmentally aged polyethylene, polyethylene terephthalate and polystyrene microplastics in aquatic environment

Recommendation: Minor Revisions

The manuscript is well supported and may be considered for publication after addressing the following points as provided below.

1. This paper should be revised to remove repetitive texts to make it more reader friendly and concise.

**Answer:**

Thank you for your insightful comment. We carefully reviewed the manuscript to identify and eliminate repetitive text throughout the manuscript. The revised version is now more concise and reader friendly, without comprising on clarity or important scientific content.

2. The authors are suggested to provide SEM images of similar magnification for easier assessment of the comparative differences.

**Answer:**

Thank you for your valuable suggestion regarding SEM image magnification. We acknowledge that presenting images at similar magnifications would facilitate direct comparison. However due to limitations during image acquisition, the SEM images were captured at different magnifications based on optimal resolutions for each samples surface morphology to clearly identify the MO dye adsorption on MPs.

This approach is consistent with previous studies such Du et al. (2022a) and Zhong et al. (2022) who also used varying magnification to effectively describe surface morphology of MPs.

3. The effect of multiple cycle of adsorption and desorption are not discussed. Authors are suggested to include long term stability test and multiple cyclic stability.

**Answer:**

We sincerely appreciate the reviewer’s insightful suggestion regarding the inclusion of long term stability and multiple adsorption desorption cycles. However the primary objective of our study was to explore the fundamental interaction mechanisms between environmentally aged PE, PET and PS MPs and the anionic dye methyl orange (MO) under varying controlling factors. As such we focused on identifying the key factors affecting adsorption and desorption rather than evaluating the long term reusability or practical application of MPs as adsorbents.

Furthermore, we note that MPs in the environment are typically regarded as contaminants themselves not as engineered adsorbent. Recent studies on dye-MPs interaction studies (Wang et al., 2023; Lin et al., 2020; Du et al 2022a, Du et al., 2022b, Zhong et al., 2022; You et al., 2021; Anastopoulos and Pashalidis, 2021) also do not emphasize cyclic stability test as their aim to understand pollutant transport potential rather than develop reusable adsorbent.

Nonetheless we acknowledge the importance of such test in studies aimed at adsorbent development. We have added this limitation and clarification to the revised manuscript and suggested that future research could address cyclic adsorption desorption stability test particularly in aged or modified MPs. (Line 501-503).

4. Presence of 16 gm/litre NaCl in simulated sea water notably decreased the desorption rate, will it be practically implementable to transfer dye into real sea water with high pH and much higher salt concentration?

**Answer:**

Thank you for highlighting this important point.

Sodium Chloride (NaCl) is widely found salt in natural environments like reservoirs, rivers, Lakes, sea, etc. One of the key elements affecting adsorption is ionic strength. In our study we used a solution containing 0-16g/L NaCl to assess the effects of ionic strength on dye absorption on MPs. However, our approach is in line with prior research (e.g., Du et al., 2022a) that also used NaCl concentrations in the range of 0-15 g/L to identify effects of ionic strength in adsorption process. Additionally, Pollutants bound to microplastics are significantly affected by NaCl, the most common ion in aqueous environments (Ma et al., 2019).

For desorption study to produce a simulated seawater a certain amount of NaCl is dissolved in distilled water to prepare a solution with an average salinity of 33.53%. We acknowledge that this setup simplifies the complexity of real seawater, which typically contains around 35 g/L total dissolved salts and a mixture of various ions (e.g., Mg^2+^, Ca^2+^, K^+^, SO_4_^2-^) as well as naturally higher pH (8.1-8.3).

While our results indicate reduced desorption behavior due to its complex ionic composition and alkaline nature. Therefore we have now noted this limitation in the revised manuscript and recommended that future studies should employ more realistic seawater analogues or real sea water samples to better evaluate the environmental desorption behavior of dyes from MPs. (Line 503-505).

5. Microplastics are a great concern for aquatic environment. How do you ensure that dye transfer into ocean using MP (without reusing it) as carrier won’t be harmful for the aquatic flora & fauna?

**Answer:**

We thank the reviewer for raising this important concern. We would like to clarify that our study does not propose the use of MPs as a carriers to transfer dyes into marine environment rather the motive of this study is to investigate the interaction between MPs and organic pollutants (e.g., dye) under different environmental conditions to better understand the potential for pollutant transport in aquatic systems.

Both MPs and synthetic dyes are recognized as harmful contaminants. Their co-occurrence in aquatic environments raises concern about the potential for MPs to act as pollutant vectors, affecting aquatic organisms (Line 50) through ingestion or leaching. Our findings aim to contribute to the understanding of such interactions which can help in assessing environmental risk and guiding future monitoring and pollution mitigation efforts.

6. Transportation of dye adsorbed MP to the sea water will be an added cost to the entire process.

Answer:

We thank the reviewer for this thoughtful observation. We would like to clarify that the aim of our study is not to propose a practical or engineering approach for dye removal via MPs followed by their transport to sea water. Rather our objectives are to simulate and understand how dyes, once absorbed onto MPs in contaminated water bodies may behave when these MPs are transported to marine environment through runoff, river or tidal exchange.

We fully agree that intentionally transporting dye –laded MPs to sea water would not be economically feasible and would raise additional environmental concern. Our study is designed to explore these interactions to better understand potential pollutant transport mechanisms that may already be occurring in real world scenario.

7. The introduction section need to be extended for importance of polymer as polymer itself is a good adsorbent. Polymer based electronic materials and their improved functionality recently inspired the researchers to choose such complex molecular system in adsorption. In the introduction section the discussion may be extended drawing inspiration from the recent reports
(i) <https://doi.org/10.1016/j.synthmet.2018.08.011>
(ii) <https://doi.org/10.1007/s00396-018-4419-3>
(iii) <https://doi.org/10.1088/2053-1591/ab12a4>
**(iv)** [**https://doi.org/10.1007/s10570-023-05286-6**](https://doi.org/10.1007/s10570-023-05286-6)

**Answer:**
We sincerely thank the reviewer for these insightful suggestions. In response we have expand the introduction section to emphasize the adsorptive potential of polymers. We have also incorporated relevant recent studies as suggested by the reviewer. (Line 69-78)

8. Adsorption and interaction of dye with MPs needs to be illustrated. Authors may visit the above references for such illustrations.

Answer:

We thank the reviewer for their constructive comment regarding the need for additional illustrations to depict the adsorption and interaction mechanisms of MO with MPs. While we appreciate the suggestion and acknowledge that visual schematics can be helpful for clarifying complex processes, we believe that our manuscript already provides a comprehensive representation of these mechanisms through several means:

(a) Detailed Discussion and Supporting Data: Our manuscript includes an extensive discussion of several adsorption process—including chemisorption, hydrogen bonding, electrostatic interactions, and π–π interactions—and is supported by experimental evidence. We present FTIR spectra that clearly demonstrate changes upon dye adsorption, SEM images illustrating surface morphology alterations, as well as adsorption kinetic data and isotherm analyses (e.g., Langmuir and Freundlich models). Together, these data effectively convey the underlying interactions without the need for additional schematic illustrations.
(b) Conciseness and Redundancy Consideration: Incorporating additional illustrations in the manuscript could lead to redundancy, as the current figures and explanations already offer a clear and detailed account of the processes under investigation. We believe that our current presentation sufficiently meets the objective of depicting the mechanisms of MO dye adsorption on aged MPs.

In light of the above, we respectfully propose that the current textual and graphical content adequately illustrates the adsorption and interaction processes of interest.

Reviewer #2: This manuscript investigates the adsorption and desorption behaviors of methyl orange (MO) dye on three types of environmentally aged microplastics (MPs): polyethylene (PE), polyethylene terephthalate (PET), and polystyrene (PS) in aquatic environments. The MPs were characterized via FTIR and SEM. Adsorption kinetics followed the pseudo-second-order model, and isotherm studies showed good fits to Langmuir and Freundlich models. Optimal removal conditions (pH 2, 27.5 mg/L MO, and 15 g/L MPs) were determined using Box-Behnken response surface methodology (RSM). Results showed that MO adsorption was affected by pH, salinity, humic acid, and MP dose. Desorption studies revealed that MO releases more readily in freshwater than in seawater, suggesting MPs could act as pollutant carriers between water systems.
The work is interesting however some minor works are required.

1. Clarity of figure no. 2 and 3 is unsatisfactory. Please provide better quality image for proper understanding.

Answer:

We thank the reviewer for their valuable comments. In the revised manuscript, we have updated Figures 2 and 3 with high-resolution versions to improve clarity and ensure that the data are represented in a more comprehensible manner.

2. In the FTIR analysis, the use of internal databases is mentioned, but no example spectra or validation of peak assignments are provided. The author is suggested to include comparison tables or spectra with annotated peaks in the supplementary.

Answer:

We thank the reviewer for highlighting these points. In methodology ( section 2.2) we clearly explain the process of ART-FTIR process as “FTIR spectra of the prepared MPs were recorded before and after adsorption of MO in an FTIR spectrometer (NICOLET IS20, Thermo Scientific, USA) in the range of 500–4,000 cm^-1^ with 16 co-scans collected at 4 cm^-1^ resolutions, and well-equipped with attenuated total reflection (ATR) units. The obtained spectra were processed with Omnic software (OMNIC 8.2) from Nicolet Instrument Corp. (Madison, WI, USA) and identified by comparing them with the internal polymer spectra library databases (Hummel polymer sample library, HR Nicolet Sampler Library, Aldrich condensed Phase Sample Library, etc).”

The Hummel Polymer, HR Nicolet Sampler, and Aldrich Condensed Phase Sample Libraries are all compatible with the Nicolet iS20 ATR-FTIR system from Thermo Scientific, but they themselves do not define the similarity metric used for identification.

Instead, the OMNIC software that runs on the Nicolet iS20 handles the spectral matching. OMNIC typically uses a Hit Quality Index (HQI) or correlation score, which may be displayed as a percentage similarity depending on your settings. If your OMNIC software is set to display match scores as percentages, then you’ll see results like “92% match to polyethylene.” Otherwise, it might show a numerical HQI (e.g., 850 out of 1000). In our study it was over 70% to confirm a specific polymer. In the supplementary information we used only representative spectra for each polymer.

In the revised manuscript, we have included the sentence “**Finally, the spectrum obtained by ATR-FTIR that matched >70 % to the reference database to confirm the specific kind of polymer**”. **(Line 133-135).**

3. There’s repetition in lines 90–91: “To assess the desorption efficiency…both freshwater and marine environments.” Please check and correct.

**Answer:**

Thank you for pointing that out. We have revised the sentence to eliminate the repetition and improve clarity. (Line 108-109)

4. In adsorption experiment part limited information on quality control/blank samples or error analysis is provided. The author is suggested to include standard deviation/error bars in figures and discuss reproducibility.

**Answer:**

Thank you for the valuable suggestion. We have revised the Methods section (Line 210-233) to include a detailed description of the quality control procedures and blank sample analyses. Additionally, all experimental data are now presented with error bars indicating standard deviations, and a discussion on reproducibility has been added.

5. The author is advised to state the rationale for selecting the specific dye concentration ranges (5–50 mg/L).

**Answer:**

Thank you for your insightful comment. The dye concentration range of 5-50 mg/L was selected based on a combination of environmentally relevant concentrations and adsorption capacity considerations found in prior studies. Lower concentration (5-10 mg/L) reflects levels commonly observed in dye contaminated effluents or aquatic systems impacted by textile wastewater allowing us to simulate realistic environmental condition. Higher concentration (up to 50 mg/L) were included to assess the adsorption performance of microplastics under condition of increased contaminant load, which are often reported in industrial discharge scenarios. Further this range falls within the optimal linear detection limits of UV-vis spectrophotometry for methyl orange. However the prior study from Zhong et. al. (2022) investigating the adsorption behaviors of malachite green (MG) and rhodamine B (RhB) on polyethylene (PE) and polyvinyl chloride (PVC) through using dye concentration 5-50 mg/L.

6. Please provide justification for 48 hours being selected as the equilibrium time (from contact time experiments).

**Answer:**
The contact time between dye molecules and MPs adsorbents (PE, PET, and PS-MPs) plays a crucial role in determining adsorption efficiency and kinetics. Equilibrium time was established through batch experiments, with contact durations ranging from **0.5 to 96 hours** at **120 rpm**.

Results indicate that Equilibrium reached at **48 hours**, as shown in **Figure 2(a)**. Further evidence suggests equilibrium times vary depending on study conditions: **48 hours (Du et al., 2022a, 2022b), 72 hours (You et al., 2021), 24 hours (Anastopoulos & Pashalidis, 2021), and 60 hours (Zhong et al., 2022).**

Thus, **48 hours was selected as the equilibrium time**, ensuring maximal adsorption while maintaining experimental efficiency.

7. In characterization part SEM micrographs are referred to but lack quantitative pore or surface roughness data. Please add porosity or surface area measurement (BET analysis), if possible.

**Answer:**

We appreciate the reviewer’s suggestion regarding the inclusion of quantitative surface characterization data. While SEM images were used to observe surface morphology and qualitative changes due to environmental aging and adsorption, we acknowledge the absence of quantitative measurements such as pore size distribution, or specific surface area (e.g., BET analysis).

Due to equipment and resource limitations at the time of the study, BET surface area analysis could not be performed. However, we recognize that such data would strengthen material characterization and offer deeper insights into the adsorption mechanism. We have now included a statement acknowledging this limitation (L 505-507) in the revised manuscript.

8. FTIR shifts after adsorption could be better explained with clear molecular interaction reasoning. Please check.

**Answer:**
Thank you for your comment. We have revised the manuscript (Line 236-247) to provide a more detailed explanation of the **FTIR peak shifts** and the associated **molecular interactions** in **Section 3.1 (Identification and Characteristics of MPs) and in Table S8 (supplementary materials).** This revision clarifies how adsorption affects functional groups, improving the interpretation of spectral changes.

9. In kinetic and isotherm modelling part, Freundlich model fit is claimed better for PS, but the paper doesn’t deeply discuss the surface heterogeneity differences between polymers. The author is suggested to discuss physicochemical structure of PS that might explain heterogeneous sites.

**Answer**:

We sincerely thank the reviewer for this insightful comment. In the revised manuscript (Line 394-398) we have expanded the disunion on the physiochemical characteristics of polystyrene (PS) that likely contribute to the heterogeneous adsorption behavior observed

The following information is included in revised manuscript

“PS structure consists of a long hydrocarbon chain with a phenyl group gives PS unique properties allow interaction with other material (Ahmed and Hashim, 2022). PS holds aromatic structure with benzene rings that promote π-π interaction with dye molecules, contributing to heterogeneous adsorption sites. Additionally environmental aging can introduce oxygen containing functional groups and surface irregularities enhancing surface heterogeneity (chen et al., 2023; Hüffer et al., 2018, Yao et al., 2023)”.

10. Tables S5 and S6 are informative but the main text lacks a clear synthesis of interaction effects beyond stating statistical values. Please add a short paragraph interpreting the interaction plots physically (e.g., "why pH and MO interact").

**Answer:**

We appreciate the reviewer’s suggestion regarding the inclusion of interaction of MO and pH from the tables S5. We have revised the manuscript and included the following information in the main text (Line 428-436).

“At low pH (2), adsorption is enhanced due to strong surface charge interactions, resulting in higher removal efficiencies (Runs 4, 15, 16, 17). At neutral pH (6.5), adsorption is moderate (Runs 1, 2, 5, 6, 8, 13) as electrostatic forces weaken, while at high pH (11), adsorption decreases significantly (Runs 3, 7, 9, 10, 11) due to repulsion effects.

The interaction between pH and MO concentration further reveals adsorption efficiency variations. At low MO concentration (5 mg/L), lower pH improves adsorption slightly (Runs 1, 4, 7, 14), while at medium concentration (27.5 mg/L), acidic conditions consistently enhance adsorption (Runs 2, 5, 6, 8, 9, 13, 17). For high MO concentrations (50 mg/L), low pH (Run 15) results in significantly greater adsorption compared to high pH (Run 3)”.

11. The composition of the "simulated seawater" is said to be in the supplementary material, but no summary or chemical composition is presented in the main text. As without knowing the exact ionic content (e.g., Na⁺, Cl⁻, Mg²⁺, SO₄²⁻), it's impossible to interpret the ionic strength or how it competes with dye desorption, it is advised to provide at least a brief table or mention of key ionic components and total ionic strength in the main text.

**Answer:**

We thank the reviewer for pointing out the need for more clarity regarding the composition of the simulates seawater. We revised the manuscript.

In our study sodium chloride (NaCl) was intentionally selected to simulate the ionic strength of sea water rather than reproducing its full ionic composition. This methodological choice aligns with several peer reviewed studies that have demonstrated the effectiveness of Nacl as a representative salt to model salinity or ionic strength effects in adsorption and desorption studies of microplastics and other contaminants.

It is true that natural seawater contains a wide variety of ions (e.g., Na⁺, Cl⁻, Mg²⁺, SO₄²⁻). In our study NaCl was used as a representative salt to simulate ionic strength. NaCl serves Na+ and Cl- accounts for over 85% of the total ionic content in the marine environment. This approach is widely adopted in adsorption and desorption studies due to its simplicity and the dominant presence of Na+ and Cl- ions in natural seawater. Using NaCl allows for controlled experimentation and facilities mechanistic interpretation, especially in assessing the effects of ionic strength on dye desorption behavior from microplastics. Environmentally aged PE and PP microplastics adsorption and desorption behavior with cationic dye crystal violet in fresh and simulated seawater with an average salinity of 33.54% (Du et al., 2022a). Another study by Shah et al., (2020) investigate the interaction between microplastics and crude oil in simulated sea water (by dissolving 350 mg NaCl into 100 Ml deionized water for final concentration in 35 ppt) and digestive tract of aquatic organism has been examined. Liu et al., (2020) systematically investigated the desorption mechanism of pharmaceuticals from pristine and aged polystyrene MPs under simulated gastrointestinal condition, Simulated seawater (550 mM NaCl and pH 7.0) was prepared for comparison.

Hence, although our simulated sea water is a simplified model, it follows widely acceptable practices in the field and provide valid insights into ionic strength effects.

12. The authors claim higher desorption in freshwater than seawater is due to "competition of NaCl for sites" — but this is unclear and counterintuitive since freshwater has lower ionic strength. Please provide necessary explanations regarding this.

**Answer:**

Thank you for your comment. The reduced desorption in seawater is not solely due to competition for binding sites but rather a combination of increased ionic strength, charge shielding, and potential salting-out effects. In seawater, the presence of high concentrations of Na⁺, Cl⁻, and other multivalent ions (Mg²⁺, Ca²⁺, SO₄²⁻) leads to the compression of the electrical double layer around both microplastic surfaces and dye molecules. This compression reduces electrostatic repulsion, thereby stabilizing the dye-polymer interaction and limiting desorption.

In contrast, freshwater has lower ionic strength, allowing more extended electrostatic interactions between dye molecules and MPs surfaces. These results in greater mobility of adsorbed dye molecules, facilitating higher desorption rates compared to seawater conditions.

13. Desorption efficiency is reported as a percentage, but no absolute desorbed amount (mg/g) is given. Please report desorbed MO in mg/g or μmol/g alongside the percentage values as % makes it difficult to directly compare desorption capacity across different MP types.

**Answer:**

Thank you for your comment. We have revised the manuscript (Line 467-469) and provide absolute amount of desorbed Methyl Orange (MO) in mg/g.

14. SEM and FTIR figures (Fig 1, S1) should be labeled more clearly with scale bars and peak labels for proper understanding.

**Answer:**

Thank you for your valuable feedback. We appreciate the reviewer suggestion. All the SEM images (S1 Fig.) already include scale bars clearly displayed at the bottom of each images for spatial references. Regarding the FTIR spectra (Fig. 1) the x axis clearly indicates the wavenumber range from 500 to 4000 cm^-1^. To further support interpretation we have included a detailed table in the supplementary information that lists the observed FTIR pecks, corresponding bonds and their associated functional groups. We hope this adequately addresses the reviewer concern and improves the clarity and interpretability of the figures.

.

15. Some references lack proper formatting, e.g., missing volume/issue/page or authors not consistently abbreviated. Please recheck all references per journal guidelines.

**Answer:**

Thank you for pointing this out. We have carefully reviewed all references and updated them to comply with Journal formatting guidelines.

16. The manuscript lacks scientific clarity due to inconsistent terminology, limited mechanistic explanation, and poor integration of results with prior studies. Additionally, redundant phrasing, unclear figure interpretation, and vague variable labeling reduce readability and hinder effective communication of findings. For example: The term “chemisorption” is used without a clear definition, while elsewhere, “electrostatic interaction” and “hydrophilic interaction” are mentioned. Lack of precise language makes mechanisms vague. Please check and correct wherever necessary.

Answer:

We sincerely thank the reviewer for highlighting the issues related to scientific clarity, terminology and mechanistic explanation. We have carefully revised the manuscript to ensure clarity in terminology, redundant phrasing and vague language are clearly explained. (Line 365-366, 289-290).

17. The authors are advised to study on the scalability of the adsorbent for practical applications at a large scale. Please check below reference.
<https://doi.org/10.1016/j.mtcomm.2024.109061>

**Answer:**

We sincerely appreciate the reviewer valuable comment regarding the scalability of the adsorbent. However we would like to clarify that the primary objective of our study was not to evaluate microplastics as a viable adsorbent for practical dye removal application but rather to investigate the adsorption and desorption behaviors of methyl Orange dye on different types of microplastics (PE, PET and PS) under different environmental factors.

Our focus was to understand how various environmental and physiochemical factors (e.g., pH, dye concentration, microplastics dose, contact time, ionic strength, natural organic matter and in natural environment) influence the adsorption interaction between MPs and dye molecules. This research aim to contribute to the growing body of knowledge on how MPs behaves as contaminant carrier in aquatic environment which is crucial for understanding their environmental risks.

It is important to emphasize that microplastics themselves are environmental contaminants and their role in pollutant adsorption is one of concern due to the potential for pollutant transport and remobilization rather their use in water purification technologies. Our studies aligns with recent research trends (Du et al 2022a, Du et al., 2022b, You et al., 2021; Zhong et al., 2022; Anastopoulos and Pashalidis, 2021; Lin et al., 2020; Wang et al., 2023)

As such scalability and regeneration studies were beyond the scope of this investigation. Nevertheless, we acknowledge the importance of such assessment for applied research and future studies may build on our findings to explore the potential of plastic based or modified materials for large scale wastewater treatment (Line 508-510).

18. Please add a section comparing performance with other published adsorbents to explain better efficiency of the present adsorbent material.

**Answer:**

Thank you for your valuable suggestion. In response, we have now included a comparative table on adsorbent efficiency in the supplementary material.

19. The author is advised to add detailed cost analysis of making the adsorbent. Please check below reference.
<https://doi.org/10.1016/j.jics.2024.101219>

Answer:

We appreciate the reviewer comment regarding the inclusion of a cost analysis for adsorbent. We would like to clarify that the primary objective of this study was not to develop or recommend MPs (PE, PET and PS) as engineered or scalable adsorbent for dye removal, rather our focus was to investigate the interaction between these widely distributed MPs and methyl orange (MO) dye under varying environmental condition to better understand their role as passive pollutant carrier in aquatic ecosystem.

As such the study does not involve any fabrication or chemical modification of the microplastics nor any synthesis process that would typically warrant a cost evaluation. We collect the adsorbent from the environment and further process for experiment. The research aim to simulate real world contamination scenario rather than propose a treatment solution.

Moreover a review of recent literature on adsorption and desorption of dye molecules on microplastics confirms that cost analysis is typically not included in such environmental behavior studies (Wang et al., 2023; Lin et al., 2020; Du et al 2022a, Du et al., 2022b, Zhong et al., 2022; You et al., 2021; Anastopoulos and Pashalidis, 2021). These studies, like ours primarily aim to assess environmental fate and interaction mechanisms rather than the practical application of MPs as cost effective adsorbent.

20. The introduction is overly broad, lacks a clear research gap, uses inconsistent flow and redundant phrasing, and fails to define key concepts like environmental aging or link the study objectives to existing literature gaps. Please check.

**Answer:**
We sincerely thank the reviewer for their insightful feedback. In response, we have substantially revised the introduction (Line 51-57, 98-101) to ensure a clear research gap, improved logical flow, and concise phrasing. Key concepts, such as environmental aging, have now been explicitly defined, and the study objectives are clearly linked to existing literature gaps to better contextualize our research. We appreciate the reviewer’s suggestion and believe these revisions enhance the clarity and relevance of the introduction.

We also have revised our manuscript accordingly, and all changes are clearly marked in the revised files.

**Comment 1: PLOS ONE Style Requirements and File Naming**

We have carefully revised the manuscript to meet all PLOS ONE style guidelines and ensured that our file names conform to the provided templates. All formatting, as well as figures and tables, have been updated accordingly.

**Comment 2: Discrepancy in Funding Information**

We noticed the discrepancies between the ‘Funding Information’ and the ‘Financial Disclosure’ sections. We have now reviewed our funding details thoroughly and updated .

**Comment 3: Funding Information Should Not Appear in the Acknowledgments**

In line with the journal’s guidelines, we have removed all funding-related text from the Acknowledgments section.

**Comment 4: Supporting Information Captions and In-Text Citations**

We have added comprehensive captions for all Supporting Information files at the end of our manuscript. Additionally, all in-text citations referring to these files have been updated to ensure consistency with the Supporting Information guidelines.

We believe these revisions have substantially improved the clarity and quality of our manuscript and have addressed all the points raised. The revised materials submitted include:

1. A separate response letter (this document).
2. A marked-up copy of the revised manuscript showing all changes labeled as 'Revised Manuscript with Track Changes’. ( In our response, text highlighted in **green** indicates our replies to Reviewer #1’s comments, and text highlighted in **yellow** indicates our replies to Reviewer #2’s comments.)
3. A clean, unmarked version of the revised manuscript.

Thank you once again for the valuable feedback. We look forward to your positive consideration.

Sincerely,

Gopal Chandra Ghosh, PhD

Professor

Department of Environmental Science and Technology

Jashore University of Science and Technology

Jashore-7408

Bangladesh

E-mail: [gopal@just.edu.bd](mailto:gopal@just.edu.bd)
