# Supplementary material for: Adsorption and desorption of methyl orange dye on environmentally aged polyethylene, polyethylene terephthalate and polystyrene microplastics in aquatic environment
Source: PLoS One. 2025 Jul 28;20(7):e0323516. doi: 10.1371/journal.pone.0323516 (PMC12303273; doi:10.1371/journal.pone.0323516)
Supplement: Peer review history

# Peer review: Decision Letter 0

**DOI:** 10.1371/journal.pone.0323516.r001

**Authors:** Amitava Mukherjee

PONE-D-25-18547Adsorption and desorption of methyl orange dye on environmentally aged polyethylene, polyethylene terephthalate and polystyrene microplastics in aquatic environmentPLOS ONE

Dear Dr. Ghosh,

Thank you for submitting your manuscript to PLOS ONE. After careful consideration, we feel that it has merit but does not fully meet PLOS ONE’s publication criteria as it currently stands. Therefore, we invite you to submit a revised version of the manuscript that addresses the points raised during the review process.

Please submit your revised manuscript by Jun 29 2025 11:59PM. If you will need more time than this to complete your revisions, please reply to this message or contact the journal office at plosone@plos.org . When you're ready to submit your revision, log on to https://www.editorialmanager.com/pone/ and select the 'Submissions Needing Revision' folder to locate your manuscript file.

Please include the following items when submitting your revised manuscript:

- A rebuttal letter that responds to each point raised by the academic editor and reviewer(s). You should upload this letter as a separate file labeled 'Response to Reviewers'.
- A marked-up copy of your manuscript that highlights changes made to the original version. You should upload this as a separate file labeled 'Revised Manuscript with Track Changes'.
- An unmarked version of your revised paper without tracked changes. You should upload this as a separate file labeled 'Manuscript'.

If you would like to make changes to your financial disclosure, please include your updated statement in your cover letter. Guidelines for resubmitting your figure files are available below the reviewer comments at the end of this letter.

If applicable, we recommend that you deposit your laboratory protocols in protocols.io to enhance the reproducibility of your results. Protocols.io assigns your protocol its own identifier (DOI) so that it can be cited independently in the future. For instructions see: https://journals.plos.org/plosone/s/submission-guidelines#loc-laboratory-protocols . Additionally, PLOS ONE offers an option for publishing peer-reviewed Lab Protocol articles, which describe protocols hosted on protocols.io. Read more information on sharing protocols at https://plos.org/protocols?utm_medium=editorial-email&utm_source=authorletters&utm_campaign=protocols .

We look forward to receiving your revised manuscript.

Kind regards,

Amitava Mukherjee, ME, Ph.D.

Academic Editor

PLOS ONE

Journal Requirements:

When submitting your revision, we need you to address these additional requirements.

1. Please ensure that your manuscript meets PLOS ONE's style requirements, including those for file naming. The PLOS ONE style templates can be found at https://journals.plos.org/plosone/s/file?id=wjVg/PLOSOne_formatting_sample_main_body.pdf and https://journals.plos.org/plosone/s/file?id=ba62/PLOSOne_formatting_sample_title_authors_affiliations.pdf

2. We note that the grant information you provided in the ‘Funding Information’ and ‘Financial Disclosure’ sections do not match.

When you resubmit, please ensure that you provide the correct grant numbers for the awards you received for your study in the ‘Funding Information’ section.

3. Thank you for stating the following in the Acknowledgments Section of your manuscript:

We thank Jashore University of Science and Technology, Bangladesh and the Ministry of Science and Technology, Bangladesh for financial support of this study. We would like to extend thanks to The World Academy of Science (TWAS) for instrumental facility under the COMSTECH-TWAS Joint Research Grants Programme (TWAS Ref: 13-371 RG/ENG/AS_C; 460 UNISCO FR: 3240279207)

We note that you have provided funding information that is not currently declared in your Funding Statement. However, funding information should not appear in the Acknowledgments section or other areas of your manuscript. We will only publish funding information present in the Funding Statement section of the online submission form.

Please remove any funding-related text from the manuscript and let us know how you would like to update your Funding Statement. Currently, your Funding Statement reads as follows:

The author(s) received no specific funding for this work.

Please include your amended statements within your cover letter; we will change the online submission form on your behalf.

4. Please include captions for your Supporting Information files at the end of your manuscript, and update any in-text citations to match accordingly. Please see our Supporting Information guidelines for more information: http://journals.plos.org/plosone/s/supporting-information.

[Note: HTML markup is below. Please do not edit.]

Reviewers' comments:

Reviewer's Responses to Questions

**Comments to the Author**

1. Is the manuscript technically sound, and do the data support the conclusions?

The manuscript must describe a technically sound piece of scientific research with data that supports the conclusions. Experiments must have been conducted rigorously, with appropriate controls, replication, and sample sizes. The conclusions must be drawn appropriately based on the data presented. 

Reviewer #1: Yes

Reviewer #2: Yes

**********

2. Has the statistical analysis been performed appropriately and rigorously? 

Reviewer #1: Yes

Reviewer #2: Yes

**********

3. Have the authors made all data underlying the findings in their manuscript fully available?

The PLOS Data policy requires authors to make all data underlying the findings described in their manuscript fully available without restriction, with rare exception (please refer to the Data Availability Statement in the manuscript PDF file). The data should be provided as part of the manuscript or its supporting information, or deposited to a public repository. For example, in addition to summary statistics, the data points behind means, medians and variance measures should be available. If there are restrictions on publicly sharing data—e.g. participant privacy or use of data from a third party—those must be specified.

Reviewer #1: Yes

Reviewer #2: Yes

**********

4. Is the manuscript presented in an intelligible fashion and written in standard English?

PLOS ONE does not copyedit accepted manuscripts, so the language in submitted articles must be clear, correct, and unambiguous. Any typographical or grammatical errors should be corrected at revision, so please note any specific errors here.

Reviewer #1: Yes

Reviewer #2: Yes

**********

5. Review Comments to the Author

Please use the space provided to explain your answers to the questions above. You may also include additional comments for the author, including concerns about dual publication, research ethics, or publication ethics. (Please upload your review as an attachment if it exceeds 20,000 characters)

Reviewer #1: Manuscript Reference: PONE-D-25-18547

Manuscript Title: Adsorption and desorption of methyl orange dye on environmentally aged polyethylene, polyethylene terephthalate and polystyrene microplastics in aquatic environment

Recommendation: Minor Revisions

The manuscript is well supported and may be considered for publication after addressing the following points as provided below.

1. This paper should be revised to remove repetitive texts to make it more reader friendly and concise.

2. The authors are suggested to provide SEM images of similar magnification for easier assessment of the comparative differences.

3. The effect of multiple cycle of adsorption and desorption are not discussed. Authors are suggested to include long term stability test and multiple cyclic stability.

4. Presence of 16 gm/litre NaCl in simulated sea water notably decreased the desorption rate, will it be practically implementable to transfer dye into real sea water with high pH and much higher salt concentration?

5. Microplastics are a great concern for aquatic environment. How do you ensure that dye transfer into ocean using MP (without reusing it) as carrier won’t be harmful for the aquatic flora & fauna?

6. Transportation of dye adsorbed MP to the sea water will be an added cost to the entire process.

7. The introduction section need to be extended for importance of polymer as polymer itself is a good adsorbent. Polymer based electronic materials and their improved functionality recently inspired the researchers to choose such complex molecular system in adsorption. In the introduction section the discussion may be extended drawing inspiration from the recent reports

(i) https://doi.org/10.1016/j.synthmet.2018.08.011

(ii) https://doi.org/10.1007/s00396-018-4419-3

(iii) https://doi.org/10.1088/2053-1591/ab12a4

(iv) https://doi.org/10.1007/s10570-023-05286-6

8. Adsorption and interaction of dye with MPs needs to be illustrated. Authors may visit the above references for such illustrations.

Reviewer #2: This manuscript investigates the adsorption and desorption behaviors of methyl orange (MO) dye on three types of environmentally aged microplastics (MPs): polyethylene (PE), polyethylene terephthalate (PET), and polystyrene (PS) in aquatic environments. The MPs were characterized via FTIR and SEM. Adsorption kinetics followed the pseudo-second-order model, and isotherm studies showed good fits to Langmuir and Freundlich models. Optimal removal conditions (pH 2, 27.5 mg/L MO, and 15 g/L MPs) were determined using Box-Behnken response surface methodology (RSM). Results showed that MO adsorption was affected by pH, salinity, humic acid, and MP dose. Desorption studies revealed that MO releases more readily in freshwater than in seawater, suggesting MPs could act as pollutant carriers between water systems.

The work is interesting however some minor works are required.

1. Clarity of figure no. 2 and 3 is unsatisfactory. Please provide better quality image for proper understanding.

2. In the FTIR analysis, the use of internal databases is mentioned, but no example spectra or validation of peak assignments are provided. The author is suggested to include comparison tables or spectra with annotated peaks in the supplementary.

3. There’s repetition in lines 90–91: “To assess the desorption efficiency…both freshwater and marine environments.” Please check and correct.

4. In adsorption experiment part limited information on quality control/blank samples or error analysis is provided. The author is suggested to include standard deviation/error bars in figures and discuss reproducibility.

5. The author is advised to state the rationale for selecting the specific dye concentration ranges (5–50 mg/L).

6. Please provide justification for 48 hours being selected as the equilibrium time (from contact time experiments).

7. In characterization part SEM micrographs are referred to but lack quantitative pore or surface roughness data. Please add porosity or surface area measurement (BET analysis), if possible.

8. FTIR shifts after adsorption could be better explained with clear molecular interaction reasoning. Please check.

9. In kinetic and isotherm modelling part, Freundlich model fit is claimed better for PS, but the paper doesn’t deeply discuss the surface heterogeneity differences between polymers. The author is suggested to discuss physicochemical structure of PS that might explain heterogeneous sites.

10. Tables S5 and S6 are informative but the main text lacks a clear synthesis of interaction effects beyond stating statistical values. Please add a short paragraph interpreting the interaction plots physically (e.g., "why pH and MO interact").

11. The composition of the "simulated seawater" is said to be in the supplementary material, but no summary or chemical composition is presented in the main text. As without knowing the exact ionic content (e.g., Na⁺, Cl⁻, Mg²⁺, SO₄²⁻), it's impossible to interpret the ionic strength or how it competes with dye desorption, it is advised to provide at least a brief table or mention of key ionic components and total ionic strength in the main text.

12. The authors claim higher desorption in freshwater than seawater is due to "competition of NaCl for sites" — but this is unclear and counterintuitive since freshwater has lower ionic strength. Please provide necessary explanations regarding this.

13. Desorption efficiency is reported as a percentage, but no absolute desorbed amount (mg/g) is given. Please report desorbed MO in mg/g or μmol/g alongside the percentage values as % makes it difficult to directly compare desorption capacity across different MP types.

14. SEM and FTIR figures (Fig 1, S1) should be labeled more clearly with scale bars and peak labels for proper understanding .

15. Some references lack proper formatting, e.g., missing volume/issue/page or authors not consistently abbreviated. Please recheck all references per journal guidelines.

16. The manuscript lacks scientific clarity due to inconsistent terminology, limited mechanistic explanation, and poor integration of results with prior studies. Additionally, redundant phrasing, unclear figure interpretation, and vague variable labeling reduce readability and hinder effective communication of findings. For example : The term “chemisorption” is used without a clear definition, while elsewhere, “electrostatic interaction” and “hydrophilic interaction” are mentioned. Lack of precise language makes mechanisms vague. Please check and correct wherever necessary.

17. The authors are advised to study on the scalability of the adsorbent for practical applications at a large scale. Please check below reference.

https://doi.org/10.1016/j.mtcomm.2024.109061

18. Please add a section comparing performance with other published adsorbents to explain better efficiency of the present adsorbent material.

19. The author is advised to add detailed cost analysis of making the adsorbent. Please check below reference.

https://doi.org/10.1016/j.jics.2024.101219

20. The introduction is overly broad, lacks a clear research gap, uses inconsistent flow and redundant phrasing, and fails to define key concepts like environmental aging or link the study objectives to existing literature gaps. Please check.

**********

6. PLOS authors have the option to publish the peer review history of their article (what does this mean? ). If published, this will include your full peer review and any attached files.

If you choose “no”, your identity will remain anonymous but your review may still be made public.

**Do you want your identity to be public for this peer review?** For information about this choice, including consent withdrawal, please see our Privacy Policy .

Reviewer #1: **Yes: ** Biswajit Saha

Reviewer #2: No

**********

[NOTE: If reviewer comments were submitted as an attachment file, they will be attached to this email and accessible via the submission site. Please log into your account, locate the manuscript record, and check for the action link "View Attachments". If this link does not appear, there are no attachment files.]

While revising your submission, please upload your figure files to the Preflight Analysis and Conversion Engine (PACE) digital diagnostic tool, https://pacev2.apexcovantage.com/ . PACE helps ensure that figures meet PLOS requirements. To use PACE, you must first register as a user. Registration is free. Then, login and navigate to the UPLOAD tab, where you will find detailed instructions on how to use the tool. If you encounter any issues or have any questions when using PACE, please email PLOS at figures@plos.org . Please note that Supporting Information files do not need this step.

# Author comment: Author response to Decision Letter 1

**DOI:** 10.1371/journal.pone.0323516.r002

19 Jun 2025

Dear Academic Editor,

Thank you for the opportunity to revise our manuscript (PONE D 25 18547) titled “Adsorption and desorption of methyl orange dye on environmentally aged polyethylene, polyethylene terephthalate and polystyrene microplastics in aquatic environment.” We appreciate the constructive feedback provided by you and the reviewers. Below we provide a point‐by‐point response to all comments raised.

Reviewer #1: Manuscript Reference: PONE-D-25-18547

Manuscript Title: Adsorption and desorption of methyl orange dye on environmentally aged polyethylene, polyethylene terephthalate and polystyrene microplastics in aquatic environment

Recommendation: Minor Revisions

The manuscript is well supported and may be considered for publication after addressing the following points as provided below.

1. This paper should be revised to remove repetitive texts to make it more reader friendly and concise.

Answer:

Thank you for your insightful comment. We carefully reviewed the manuscript to identify and eliminate repetitive text throughout the manuscript. The revised version is now more concise and reader friendly, without comprising on clarity or important scientific content.

2. The authors are suggested to provide SEM images of similar magnification for easier assessment of the comparative differences.

Answer:

Thank you for your valuable suggestion regarding SEM image magnification. We acknowledge that presenting images at similar magnifications would facilitate direct comparison. However due to limitations during image acquisition, the SEM images were captured at different magnifications based on optimal resolutions for each samples surface morphology to clearly identify the MO dye adsorption on MPs.

This approach is consistent with previous studies such Du et al. (2022a) and Zhong et al. (2022) who also used varying magnification to effectively describe surface morphology of MPs.

3. The effect of multiple cycle of adsorption and desorption are not discussed. Authors are suggested to include long term stability test and multiple cyclic stability.

Answer:

We sincerely appreciate the reviewer’s insightful suggestion regarding the inclusion of long term stability and multiple adsorption desorption cycles. However the primary objective of our study was to explore the fundamental interaction mechanisms between environmentally aged PE, PET and PS MPs and the anionic dye methyl orange (MO) under varying controlling factors. As such we focused on identifying the key factors affecting adsorption and desorption rather than evaluating the long term reusability or practical application of MPs as adsorbents.

Furthermore, we note that MPs in the environment are typically regarded as contaminants themselves not as engineered adsorbent. Recent studies on dye-MPs interaction studies (Wang et al., 2023; Lin et al., 2020; Du et al 2022a, Du et al., 2022b, Zhong et al., 2022; You et al., 2021; Anastopoulos and Pashalidis, 2021) also do not emphasize cyclic stability test as their aim to understand pollutant transport potential rather than develop reusable adsorbent.

Nonetheless we acknowledge the importance of such test in studies aimed at adsorbent development. We have added this limitation and clarification to the revised manuscript and suggested that future research could address cyclic adsorption desorption stability test particularly in aged or modified MPs. (Line 501-503).

4. Presence of 16 gm/litre NaCl in simulated sea water notably decreased the desorption rate, will it be practically implementable to transfer dye into real sea water with high pH and much higher salt concentration?

Answer:

Thank you for highlighting this important point.

Sodium Chloride (NaCl) is widely found salt in natural environments like reservoirs, rivers, Lakes, sea, etc. One of the key elements affecting adsorption is ionic strength. In our study we used a solution containing 0-16g/L NaCl to assess the effects of ionic strength on dye absorption on MPs. However, our approach is in line with prior research (e.g., Du et al., 2022a) that also used NaCl concentrations in the range of 0-15 g/L to identify effects of ionic strength in adsorption process. Additionally, Pollutants bound to microplastics are significantly affected by NaCl, the most common ion in aqueous environments (Ma et al., 2019).

For desorption study to produce a simulated seawater a certain amount of NaCl is dissolved in distilled water to prepare a solution with an average salinity of 33.53%. We acknowledge that this setup simplifies the complexity of real seawater, which typically contains around 35 g/L total dissolved salts and a mixture of various ions (e.g., Mg2+, Ca2+, K+, SO42-) as well as naturally higher pH (8.1-8.3).

While our results indicate reduced desorption behavior due to its complex ionic composition and alkaline nature. Therefore we have now noted this limitation in the revised manuscript and recommended that future studies should employ more realistic seawater analogues or real sea water samples to better evaluate the environmental desorption behavior of dyes from MPs. (Line 503-505).

5. Microplastics are a great concern for aquatic environment. How do you ensure that dye transfer into ocean using MP (without reusing it) as carrier won’t be harmful for the aquatic flora & fauna?

Answer:

We thank the reviewer for raising this important concern. We would like to clarify that our study does not propose the use of MPs as a carriers to transfer dyes into marine environment rather the motive of this study is to investigate the interaction between MPs and organic pollutants (e.g., dye) under different environmental conditions to better understand the potential for pollutant transport in aquatic systems.

Both MPs and synthetic dyes are recognized as harmful contaminants. Their co-occurrence in aquatic environments raises concern about the potential for MPs to act as pollutant vectors, affecting aquatic organisms (Line 50) through ingestion or leaching. Our findings aim to contribute to the understanding of such interactions which can help in assessing environmental risk and guiding future monitoring and pollution mitigation efforts.

6. Transportation of dye adsorbed MP to the sea water will be an added cost to the entire process.

Answer:

We thank the reviewer for this thoughtful observation. We would like to clarify that the aim of our study is not to propose a practical or engineering approach for dye removal via MPs followed by their transport to sea water. Rather our objectives are to simulate and understand how dyes, once absorbed onto MPs in contaminated water bodies may behave when these MPs are transported to marine environment through runoff, river or tidal exchange.

We fully agree that intentionally transporting dye –laded MPs to sea water would not be economically feasible and would raise additional environmental concern. Our study is designed to explore these interactions to better understand potential pollutant transport mechanisms that may already be occurring in real world scenario.

7. The introduction section need to be extended for importance of polymer as polymer itself is a good adsorbent. Polymer based electronic materials and their improved functionality recently inspired the researchers to choose such complex molecular system in adsorption. In the introduction section the discussion may be extended drawing inspiration from the recent reports

(i) https://doi.org/10.1016/j.synthmet.2018.08.011

(ii) https://doi.org/10.1007/s00396-018-4419-3

(iii) https://doi.org/10.1088/2053-1591/ab12a4

(iv) https://doi.org/10.1007/s10570-023-05286-6

Answer:

We sincerely thank the reviewer for these insightful suggestions. In response we have expand the introduction section to emphasize the adsorptive potential of polymers. We have also incorporated relevant recent studies as suggested by the reviewer. (Line 69-78)

8. Adsorption and interaction of dye with MPs needs to be illustrated. Authors may visit the above references for such illustrations.

Answer:

We thank the reviewer for their constructive comment regarding the need for additional illustrations to depict the adsorption and interaction mechanisms of MO with MPs. While we appreciate the suggestion and acknowledge that visual schematics can be helpful for clarifying complex processes, we believe that our manuscript already provides a comprehensive representation of these mechanisms through several means:

(a) Detailed Discussion and Supporting Data: Our manuscript includes an extensive discussion of several adsorption process—including chemisorption, hydrogen bonding, electrostatic interactions, and π–π interactions—and is supported by experimental evidence. We present FTIR spectra that clearly demonstrate changes upon dye adsorption, SEM images illustrating surface morphology alterations, as well as adsorption kinetic data and isotherm analyses (e.g., Langmuir and Freundlich models). Together, these data effectively convey the underlying interactions without the need for additional schematic illustrations.

(b) Conciseness and Redundancy Consideration: Incorporating additional illustrations in the manuscript could lead to redundancy, as the current figures and explanations already offer a clear and detailed account of the processes under investigation. We believe that our current presentation sufficiently meets the objective of depicting the mechanisms of MO dye adsorption on aged MPs.

In light of the above, we respectfully propose that the current textual and graphical content adequately illustrates the adsorption and interaction processes of interest.

Reviewer #2: This manuscript investigates the adsorption and desorption behaviors of methyl orange (MO) dye on three types of environmentally aged microplastics (MPs): polyethylene (PE), polyethylene terephthalate (PET), and polystyrene (PS) in aquatic environments. The MPs were characterized via FTIR and SEM. Adsorption kinetics followed the pseudo-second-order model, and isotherm studies showed good fits to Langmuir and Freundlich models. Optimal removal conditions (pH 2, 27.5 mg/L MO, and 15 g/L MPs) were determined using Box-Behnken response surface methodology (RSM). Results showed that MO adsorption was affected by pH, salinity, humic acid, and MP dose. Desorption studies revealed that MO releases more readily in freshwater than in seawater, suggesting MPs could act as pollutant carriers between water systems.

The work is interesting however some minor works are required.

1. Clarity of figure no. 2 and 3 is unsatisfactory. Please provide better quality image for proper understanding.

Answer:

We thank the reviewer for their valuable comments. In the revised manuscript, we have updated Figures 2 and 3 with high-resolution versions to improve clarity and ensure that the data are represented in a more comprehensible manner.

2. In the FTIR analysis, the use of internal databases is mentioned, but no example spectra or validation of peak assignments are provided. The author is suggested to include comparison tables or spectra with annotated peaks in the supplementary.

Answer:

We thank the reviewer for highlighting these points. In methodology ( section 2.2) we clearly explain the process of ART-FTIR process as “FTIR spectra of the prepared MPs were recorded before and after adsorption of MO in an FTIR spectrometer (NICOLET IS20, Thermo Scientific, USA) in the range of 500–4,000 cm-1 with 16 co-scans collected at 4 cm-1 resolutions, and well-equipped with attenuated total reflection (ATR) units. The obtained spectra were processed with Omnic software (OMNIC 8.2) from Nicolet Instrument Corp. (Madison, WI, USA) and identified by comparing them with the internal polymer spectra library databases (Hummel polymer sample library, HR Nicolet Sampler Library, Aldrich condensed Phase Sample Library, etc).”

The Hummel Polymer, HR Nicolet Sampler, and Aldrich Condensed Phase Sample Libraries are all compatible with the Nicolet iS20 ATR-FTIR system from Thermo Scientific, but they themselves do not define the similarity metric used for identification.

Instead, the OMNIC software that runs on the Nicolet iS20 handles the spectral matching. OMNIC typically uses a Hit Quality Index (HQI) or correlation score, which may be displayed as a percentage similarity depending on your settings. If your OMNIC software is set to display match scores as percentages, then you’ll see results like “92% match to polyethylene.” Otherwise, it might show a numerical HQI (e.g., 850 out of 1000). In our study it was over 70% to confirm a specific polymer. In the supplementary information we used only representative spectra for each polymer.

In the revised manuscript, we have included the sentence “Finally, the spectrum obtained by ATR-FTIR that matched >70 % to the reference database to confirm the specific kind of polymer”. (Line 133-135).

3. There’s repetition in lines 90–91: “To assess the desorption efficiency…both freshwater and marine environments.” Please check and correct.

Answer:

Thank you for pointing that out. We have revised the sentence to eliminate the repetition and improve clarity. (Line 108-109)

4. In adsorption experiment part limited information on quality control/blank samples or error analysis is provided. The author is suggested to include standard deviation/error bars in figures and discuss reproducibility.

Answer:

Thank you for the valuable suggestion. We have revised the Methods section (Line 210-233) to include a detailed description of the quality control procedures and blank sample analyses. Additionally, all experimental data are now presented with error bars indicating standard deviations, and a discussion on reproducibility has been added.

5. The author is advised to state the rationale for selecting the specific dye concentration ranges (5–50 mg/L).

Answer:

Thank you for your insightful comment. The dye concentration range of 5-50 mg/L was selected based on a combination of environmentally relevant concentrations and adsorption capacity considerations found in prior studies. Lower concentration (5-10 mg/L) reflects levels commonly observed in dye contaminated effluents or aquatic systems impacted by textile wastewater allowing us to simulate realistic environmental condition. Higher concentration (up to 50 mg/L) were included to assess the adsorption performance of microplastics under condition of increased contaminant load, which are often reported in industrial discharge scenarios. Further this range falls within the optimal linear detection limits of UV-vis spectrophotometry for methyl orange. However the prior study from Zhong et. al. (2022) investigating the adsorption behaviors of malachite green (MG) and rhodamine B (RhB) on polyethylene (PE) and polyvinyl chloride (PVC) through using dye concentration 5-50 mg/L.

6. Please provide justification for 48 hours being selected as the equilibrium time (from contact time experiments).

Answer:

The contact time between dye molecules and MPs adsorbents (PE, PET, and PS-MPs) plays a crucial role in determining adsorption efficiency and kinetics. Equilibrium time was established through batch experiments, with contact durations ranging from 0.5 to 96 hours at 120 rpm.

Results indicate that Equilibrium reached at 48 hours, as shown in Figure 2(a). Further evidence suggests equilibrium times vary depending on study conditions: 48 hours (Du et al., 2022a, 2022b), 72 hours (You et al., 2021), 24 hours (Anastopoulos & Pashalidis, 2021), and 60 hours (Zhong et al., 2022).

Thus, 48 hours was selected as the equilibrium time, ensuring maximal adsorption while maintaining experimental efficiency.

7. In characterization part SEM micrographs are referred to but lack quantitative pore or surface roughness data. Please add porosity or surface area measurement (BET analysis), if possible.

Answer:

We appreciate the reviewer’s suggestion regarding the inclusion of quantitative surface characterization data. While SEM images were used to observe surface morphology and qualitative changes due to environmental aging and adsorption, we acknowledge the absence of quantitative measurements such as pore size distribution, or specif

Attachment Submitted filename: Response to Reviewers Letter.docx

# Peer review: Decision Letter 1

**DOI:** 10.1371/journal.pone.0323516.r003

**Authors:** Amitava Mukherjee

Adsorption and desorption of methyl orange dye on environmentally aged polyethylene, polyethylene terephthalate and polystyrene microplastics in aquatic environment

PONE-D-25-18547R1

Dear Dr. Ghosh,

We’re pleased to inform you that your manuscript has been judged scientifically suitable for publication and will be formally accepted for publication once it meets all outstanding technical requirements.

Within one week, you’ll receive an e-mail detailing the required amendments. When these have been addressed, you’ll receive a formal acceptance letter and your manuscript will be scheduled for publication.

An invoice will be generated when your article is formally accepted. Please note, if your institution has a publishing partnership with PLOS and your article meets the relevant criteria, all or part of your publication costs will be covered. Please make sure your user information is up-to-date by logging into Editorial Manager at Editorial Manager®  and clicking the ‘Update My Information' link at the top of the page. If you have any questions relating to publication charges, please contact our Author Billing department directly at authorbilling@plos.org.

If your institution or institutions have a press office, please notify them about your upcoming paper to help maximize its impact. If they’ll be preparing press materials, please inform our press team as soon as possible -- no later than 48 hours after receiving the formal acceptance. Your manuscript will remain under strict press embargo until 2 pm Eastern Time on the date of publication. For more information, please contact onepress@plos.org.

Kind regards,

Amitava Mukherjee, ME, Ph.D.

Academic Editor

PLOS ONE

Additional Editor Comments (optional):

Reviewers' comments:

Reviewer's Responses to Questions

**Comments to the Author**

1. If the authors have adequately addressed your comments raised in a previous round of review and you feel that this manuscript is now acceptable for publication, you may indicate that here to bypass the “Comments to the Author” section, enter your conflict of interest statement in the “Confidential to Editor” section, and submit your "Accept" recommendation.

Reviewer #2: (No Response)

**********

2. Is the manuscript technically sound, and do the data support the conclusions?

The manuscript must describe a technically sound piece of scientific research with data that supports the conclusions. Experiments must have been conducted rigorously, with appropriate controls, replication, and sample sizes. The conclusions must be drawn appropriately based on the data presented. 

Reviewer #2: (No Response)

**********

3. Has the statistical analysis been performed appropriately and rigorously? 

Reviewer #2: (No Response)

**********

4. Have the authors made all data underlying the findings in their manuscript fully available?

The PLOS Data policy requires authors to make all data underlying the findings described in their manuscript fully available without restriction, with rare exception (please refer to the Data Availability Statement in the manuscript PDF file). The data should be provided as part of the manuscript or its supporting information, or deposited to a public repository. For example, in addition to summary statistics, the data points behind means, medians and variance measures should be available. If there are restrictions on publicly sharing data—e.g. participant privacy or use of data from a third party—those must be specified.

Reviewer #2: (No Response)

**********

5. Is the manuscript presented in an intelligible fashion and written in standard English?

PLOS ONE does not copyedit accepted manuscripts, so the language in submitted articles must be clear, correct, and unambiguous. Any typographical or grammatical errors should be corrected at revision, so please note any specific errors here.

Reviewer #2: (No Response)

**********

6. Review Comments to the Author

Please use the space provided to explain your answers to the questions above. You may also include additional comments for the author, including concerns about dual publication, research ethics, or publication ethics. (Please upload your review as an attachment if it exceeds 20,000 characters)

Reviewer #2: (No Response)

**********

7. PLOS authors have the option to publish the peer review history of their article (what does this mean? ). If published, this will include your full peer review and any attached files.

If you choose “no”, your identity will remain anonymous but your review may still be made public.

**Do you want your identity to be public for this peer review?** For information about this choice, including consent withdrawal, please see our Privacy Policy .

Reviewer #2: **Yes: ** Animesh Debnath

**********

# Editor report: Acceptance letter

**DOI:** 10.1371/journal.pone.0323516.r004

**Authors:** Amitava Mukherjee

PONE-D-25-18547R1

PLOS ONE

Dear Dr. Ghosh,

I'm pleased to inform you that your manuscript has been deemed suitable for publication in PLOS ONE. Congratulations! Your manuscript is now being handed over to our production team.

At this stage, our production department will prepare your paper for publication. This includes ensuring the following:

* All references, tables, and figures are properly cited

* All relevant supporting information is included in the manuscript submission,

* There are no issues that prevent the paper from being properly typeset

You will receive further instructions from the production team, including instructions on how to review your proof when it is ready. Please keep in mind that we are working through a large volume of accepted articles, so please give us a few days to review your paper and let you know the next and final steps.

Lastly, if your institution or institutions have a press office, please let them know about your upcoming paper now to help maximize its impact. If they'll be preparing press materials, please inform our press team within the next 48 hours. Your manuscript will remain under strict press embargo until 2 pm Eastern Time on the date of publication. For more information, please contact onepress@plos.org.

If we can help with anything else, please email us at customercare@plos.org.

Thank you for submitting your work to PLOS ONE and supporting open access.

Kind regards,

PLOS ONE Editorial Office Staff

on behalf of

Professor Dr. Amitava Mukherjee

Academic Editor

PLOS ONE
